# Automated classification of tailed bacteriophages according to their neck organization

**DOI:** 10.1186/1471-2164-15-1027

**Published:** 2014-11-27

**Authors:** Anne Lopes, Paulo Tavares, Marie-Agnès Petit, Raphaël Guérois, Sophie Zinn-Justin

**Affiliations:** CEA, iBiTecS, Gif-sur-Yvette F-91191 Paris, France; Université Paris-Sud & CNRS, UMR 8221, Gif-sur-Yvette, F-91191 Paris, France; Institut de Génétique et Microbiologie, Université Paris-Sud, Orsay, Paris, France; Unité de Virologie Moléculaire et Structurale, UMR CNRS 2472, UMR INRA 1157 and IFR 115, Gif-sur-Yvette, Paris, France; INRA, UMR1319, Micalis, domaine de Vilvert, Jouy en Josas, Paris, France; AgroParisTech, UMR1319, Micalis, domaine de Vilvert, Jouy en Josas, Paris, France

**Keywords:** Bacteriophage, Profile-profile comparison, Virion, Gene organization, Evolution

## Abstract

**Background:**

The genetic diversity observed among bacteriophages remains a major obstacle for the identification of homologs and the comparison of their functional modules. In the structural module, although several classes of homologous proteins contributing to the head and tail structure can be detected, proteins of the head-to-tail connection (or neck) are generally more divergent. Yet, molecular analyses of a few tailed phages belonging to different morphological classes suggested that only a limited number of structural solutions are used in order to produce a functional virion. To challenge this hypothesis and analyze proteins diversity at the virion neck, we developed a specific computational strategy to cope with sequence divergence in phage proteins. We searched for homologs of a set of proteins encoded in the structural module using a phage learning database.

**Results:**

We show that using a combination of iterative profile-profile comparison and gene context analyses, we can identify a set of head, neck and tail proteins in most tailed bacteriophages of our database. Classification of phages based on neck protein sequences delineates 4 Types corresponding to known morphological subfamilies. Further analysis of the most abundant Type 1 yields 10 Clusters characterized by consistent sets of head, neck and tail proteins. We developed Virfam, a webserver that automatically identifies proteins of the phage head-neck-tail module and assign phages to the most closely related cluster of phages. This server was tested against 624 new phages from the NCBI database. 93% of the tailed and unclassified phages could be assigned to our head-neck-tail based categories, thus highlighting the large representativeness of the identified virion architectures. Types and Clusters delineate consistent subgroups of *Caudovirales*, which correlate with several virion properties.

**Conclusions:**

Our method and webserver have the capacity to automatically classify most tailed phages, detect their structural module, assign a function to a set of their head, neck and tail genes, provide their morphologic subtype and localize these phages within a “head-neck-tail” based classification. It should enable analysis of large sets of phage genomes. In particular, it should contribute to the classification of the abundant unknown viruses found on assembled contigs of metagenomic samples.

**Electronic supplementary material:**

The online version of this article (doi:10.1186/1471-2164-15-1027) contains supplementary material, which is available to authorized users.

## Background

Bacteriophages, which are defined as viruses that infect and replicate within bacteria, constitute the largest known group of viruses [1, 2]. They occur everywhere in the biosphere where bacteria are found, their habitats being as diverse as oceans, topsoils, plants or animals. The total number of phage species is estimated to reach millions [3, 4], and their classification remains a challenge nowadays. Whereas molecular biology has permitted for most living organisms a progressive shift from character-based classifications to classifications based on genetic markers [5], no satisfying method for classifying phages exists at present. Phages were initially sorted according to two characters: the nature of their encapsidated nucleic acid and their virion morphology. More than 96% of them are tailed phages: they constitute the *Caudovirales* order [6]. They encapsidate double stranded DNA genomes. Moreover, their viral particle is formed by a head, mainly constituted by an icosahedral capsid that protects the viral genome, and a tail specialized in DNA delivery inside the bacterial host. *Caudovirales* are divided into *Siphoviridae*, *Myoviridae* and *Podoviridae* families depending on the nature of their tail, which is respectively long and non-contractile, long and contractile, or short. Phenotypic observation does not permit finer grained structural distinction among these tailed phages, so that molecular tools are needed to further classify them [7]. At this family level, some classification problems arise, as some phages can be separated into *Sipho*-, *Myo-* and *Podoviridae*, while having closely related genomes [8, 9].

Phage hierarchical trees were obtained from the analysis of capsid proteins [10]. Terminase [11] and portal [12] proteins were further described as potential markers of phage diversity, as well as tape measure proteins in long-tail bacteriophages [13]. However, the selected gene is sometimes not detected in some of the phages, thus excluding these phages from the classification [12, 13] and limiting the use of the gene as a marker for biodiversity studies [8]. Moreover, a single gene does not provide a global view of the virion architecture, thus hindering phage classification as a function of a general virion structural organisation.

Phage hierarchical trees were recalculated based on whole genome analyses [9, 14]. To some extent this approach permitted the definition of some genera and subfamilies among *Myo-* and *Podoviridae*, but it seemed unsuccessful with respect to *Siphoviridae*. Decisive in such approaches is the capacity to group together large enough sets of orthologs, which remains difficult given the remarkable level of sequence divergence between phage proteins. Not only do phage genomes mutate more rapidly than bacterial genomes, but they also recombine more readily, so that the notions of phage species and hierarchical classification can be questioned. Using a similar estimate of relatedness based on the amount of shared genes, but departing from the hierarchical classification, an attempt at representing phage relatedness with graph theory revealed a densely connected network of all *Caudovirales*[15]. However, it is difficult to extract from such an analysis the relationship between phage classification and specific phage properties as for example virion morphology. In order to understand how highly divergent phages encode for a virion capable of infecting bacteria, we focused the analysis on a set of genes belonging to the “structural” module involved in virion assembly and host infection.

**Figure 1:**
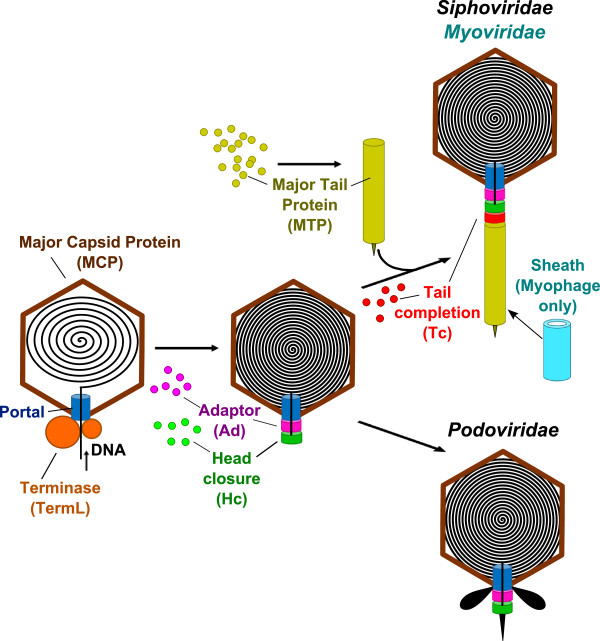
**Assembly pathway of tailed bacteriophages.** In the tailed phages, capsid assembly starts with the construction of an icosahedral protein lattice called *procapsid,* essentially composed of a *major capsid protein* (noted MCP in brown in Figure 1)*.* At a specialized vertex of the procapsid, the dodecameric *portal protein* (Portal in blue) forms a channel which is the docking point for an ATPase complex called *terminase*. This complex normally contains multiple copies of a large subunit with ATPase and endonuclease activities (TermL in orange), and a small DNA binding subunit that recognizes the cognate viral DNA Sun *et al.*[20]. It translocates viral dsDNA into the procapsid cavity through the portal channel. When DNA packaging is completed, the terminase motor disassembles and the portal dodecamer recruits *head-completion proteins* to prevent leakage of the viral DNA. One such protein directly binds to the portal: it is called the *adaptor protein* (Ad in magenta); it can also be supplemented with the so-called *head-closure protein* (Hc in green) [18, 21–23]. Altogether the head-completion proteins provide a platform for completion of short tail assembly in *Podoviridae*[24, 25] as well as for docking of pre-assembled long tails in *Sipho-* and *Myoviridae*[26–28]. Located at one end of the *Sipho-* and *Myoviridae* long tails, the *tail-completion protein* (Tc in red) allows for the tail attachment to the head. Head- and tail-completion proteins form the head-to-tail connection and, together with the portal protein, constitute the virion’s *neck*. The *major tail protein* (MTP in kaki) is the main component of the tail tube structure. In *Myoviridae*, the surrounding *tail sheath* protein (Sheath in cyan) contracts upon host infection, initiating viral DNA injection in the host cell.

Despite phage divergent and complex evolution, the thorough molecular analysis of a few paradigmatic tailed phages belonging to different morphological classes, such as *Siphoviridae* SPP1 and λ, *Myoviridae* T4 and *Podoviridae* P22 and Φ29, suggested that only a limited number of structural solutions are used in order to produce a functional virion [16–19]. To challenge this hypothesis, we searched for homologs of a set of virion proteins functionally characterized through the study of the assembly pathway of the corresponding phages (Figure 1, Table 1 and Experimental procedures). Protein names sometimes differ for the various model phages that were studied, and are unified in Figure 1 for the sake of clarity. Proteins from the head (Major Capsid Protein or MCP, portal and terminase) and the tail (Major Tail Protein or MTP, sheath) of bacteriophages are generally well conserved, and could be detected with standard bioinformatics strategies. In contrast, proteins lying at the interface between the head and tail components, the so-called Ad, Hc and Tc head-to-tail connection proteins (see Figure 1 for definitions), can be much more difficult to detect due to drastic sequence divergence.

**Table 1:** **Functionally characterized head- and tail-completion proteins of tailed bacteriophages classified in Aclame**

Phage	Order	Head-completion Ad	Head-completion Hc	Tail-completion Tc	Neck type
SPP1	*Siphoviridae*	**gp15** ^**1**^	**gp16** ^**2**^	**gp17** ^**3**^	1
Lambda	*Siphoviridae*	**gpW**	**gpFII** ^**2**^	**gpU** ^**3**^	1
HK97	*Siphoviridae*	**gp6** ^**1**^	gp7	gp9*	1
T4	*Myoviridae*	gp13	gp14	**gp15** ^**3**^	2
P22	*Podoviridae*	**gp4** ^**1**^	gp10	-	3
Φ29	*Podoviridae*	gp11	-	-	4

*Refers to a protein identified through this study**.**

Head- and tail-completion proteins extracted from the literature are listed in this table. Proteins of known 3D structure are indicated in bold. Structural homologies between proteins that were experimentally demonstrated are indicated by similar numbers in superscript. Neck types result from the current analysis. In particular, SPP1, λ and HK97 are gathered within the same phage type because they are predicted as exhibiting homologous head- and tail completion proteins. GpW of *Siphoviridae* phage λ corresponds to a rare alternative playing the role of SPP1 gp15 at the head-to-tail connection (although gpW and gp15 share no homology at the sequence and structural levels).

To obtain a global view of the “structural module” in bacteriophages and probe whether newly sequenced phages can be assigned to already known systems, we developed a specific computational strategy able to cope with the high divergence and plasticity of phage genomes. A sensitivity-enhanced bioinformatics approach based on profile-profile comparisons was initially used [29]. We further improved this method by performing systematic gene context analyses and successfully detected the head-to-tail connection proteins in 91% of 328 genomes of tailed and unclassified phages. Based on the occurrence of these proteins, phages were classified into 4 Types and their relationships with known morphological subfamilies (*Siphoviridae*, *Myoviridae*, and *Podoviridae)* were defined. Next, a similarity metric between phages, combining profile-profile comparison scores with sequence identities, was developed to provide a finer classification of virion architectures within every Type. For the latter step, not only proteins from the head-to-tail connection (neck) were considered but also components of the head and the tail. As a result, the most abundant Type 1 could be divided into 10 Clusters, some exclusively containing *Siphoviridae* or *Myoviridae*, and others aggregating both *Sipho-* and *Myoviridae*. We developed a Webserver, called Virfam, to map any novel phage genome on our classification scheme. By testing this server on 624 phage genomes from the NCBI database not yet included in our dataset, we confirmed the large applicability of our classification approach, as 93% of them could be classified within one of the Type/Cluster classes. Beyond phage classification, this study also provides insights into the possible organization of ancestral neck modules in *Caudovirales.*

## Implementation

### HMM Profiles generation for proteins of the learning database

For all the 28300 phage sequences contained in the Aclame database (version 0.4) [30], a profile was built following the protocol described in [29]. In the latest release of Aclame (version 0.4), 465 bacterial phage genomes are accessible but only 447 fulfilled the criteria of being bacteriophages (10 are phages infecting Archeae) with treatable sequences (8 were not properly implemented in Aclame). Briefly, for every sequence, a 3 iteration PSI-Blast [31] search was performed against the non-redundant (nr70 (dec. 2010)) database using a cutoff e-value of 10^-4^. Whenever an iteration retrieved more than 1000 homologs, the previous iteration was kept so as to prevent divergence issues. The resulting multiple sequence alignment was filtered so as to keep the 100 most diverse sequences and was converted into a Hidden-Markov Model profile using the HHsuite programs [32] integrating the secondary structure prediction from Psi-pred [33]. The detailed bioinformatics procedure described below was followed for each of the different component of the head-neck-tail module. To evaluate the sensitivity gains provided by profile-profile comparisons (HHsuite) with respect to profile-sequence ones (PSI-Blast), as presented in Table 2, we followed the procedure described in Additional file 1: Method S1.

**Table 2:** **Detected head- and tail-completion proteins of Aclame**

	Detected with Psi-Blast	Detected with HHsearch at 90%	Detected only at 70% using a combined approach	Percentage of proteins detected with the combined approach	Exceptions	Total protein number
Ad1	86	191 *	10	5	12	213 *
Hc1	71	158	4	2	5	167
Tc1	41	166	27	13	14	207
Ad2	17	17	0	0	0	17
Hc2	16	16	0	0	0	16
Tc2	16	16	0	0	0	16
Ad3	8	8	30	60	12	50
Hc3	17	51	0	0	0	51
Ad4	10	10	0	0	0	10
Total	282	633	71	10	43	747

*Including 5 gpW-like Ad1.

Head- and tail-completion proteins detected using either the profile-sequence comparison tool PSI-BLAST, the profile-profile comparison tool HHsearch with a threshold of 90% or an approach combining the HHsearch tool at a threshold of 70% with analysis of the gene contexts are detailed (percentages correspond to HHsearch confidence thresholds). Proteins counted in the “Exceptions” column correspond to proteins that were manually detected, either because their HMM profile was built from very few sequences (hindering profile-profile comparisons) but they showed consistent secondary structure prediction and genome localisation, or because they were detected by HHsearch but their genome positioning slightly deviated from the canonical positioning. The Aclame references of the detected neck proteins, as well as of the identified head and tail proteins, are displayed in Additional file 2: Table S1.

### Detection of the head and tail proteins within the learning database

We first searched for the major capsid proteins, portal proteins, terminases, major tail proteins and Sheath proteins within Aclame 0.4. The various components of the head and the tail were identified starting from proteins whose 3D structures were solved and using our iterative profile-profile comparison procedure with a probability confidence threshold of 90% as described in [29]. In the case of the major capsid proteins, the 3D structures are known from X-ray data for the Siphophage HK97 and the Myophage T4. The HK97 and T4 major capsid proteins exhibit a similar polypeptide fold [34]. Consistently, they were detected as related by HHsearch with a confidence score of 93%. We used their profiles as starting points in order to detect other proteins belonging to the major capsid superfamily. 290 major capsid proteins were identified, including the capsid proteins of the Siphophages SPP1, λ, Φϵ125, the Myophages P2, Mu and the Podophages T3, T7, ϵ15, P22, PZA. For several of these phages, a high resolution EM structure of the capsid is available, and the EM data are consistent with the existence of a common major capsid protein fold [35–38]. In the case of the portal proteins, the 3D structures are known for phages Φ29, SPP1 and P22. The portal proteins of these phages exhibit a common fold [39, 40]. We used their profiles as starting seeds and identified 308 portal proteins, including those of phages HK97, λ, Φϵ125, T4, P2, Mu, T3, T7 and ϵ15. 3D structures of terminase large subunits are known for phages T4 (whole gp17 protein [41] and nuclease domain [42]) and SPP1 (nuclease domain [43]). Here again, despite a very low sequence identity, the fold of the nuclease domain is conserved. Using the profiles of the SPP1 and T4 terminases as starting point, 307 large terminases were identified, including those of phages HK97, λ, Φϵ125, P2, Mu, T3, T7, P22 and ϵ15. We did not find the terminase of Φ29, consistent with a previous bioinformatics study that clearly demonstrated the existence of two structurally distinct families of large terminases, one of these families being found in most tailed bacteriophages, and the other family being only found in Φ29-like phages [44]. In the case of the major tail proteins, only one 3D structure is available, that of gpV from phage λ [19], from which we detected 185 tail proteins, including those of the Siphophage SPP1 and the Myophage P2. However, we did not identify major tail proteins for T4-like Myophages. Thus, we also searched homologs for the well-characterized T4 major tail protein gp19 and found 16 additional homologous tail proteins. Search for the fold of these proteins using HHpred [45] against a profile database derived from the Protein Data Bank revealed that T4-like MTP likely share the fold of phage λ major tail protein gpV (probability score higher than 90%). This suggests that myophages of the T4 family also present a tail protein evolutionary linked to that of the siphophages. Finally, regarding Sheath proteins, the 3D structures are known for phages T4 [46] and PhiKZ [47]. These structures exhibit a common structural core [47] that was not detected by HHsearch. 70 Sheath proteins were identified starting from the T4 protein, out of the 74 Myophages in Aclame. Only 2 Sheath proteins were identified starting from the PhiKZ protein, corresponding to the giant phages PhiKZ and PhiEL. Within the 2 myophages left without any assigned Sheath protein, C-st was experimentally shown to be a myophage and should have a Sheath protein. On the opposite, P4 is a satellite bacteriophage coding for 14 proteins, which consistently lacks a Sheath protein. At this stage, we identified head, neck and tail proteins within most of the 447 phages of Aclame.

### Detection of the head- and tail-completion proteins using remote homology search and genetic context

**Figure 2:**
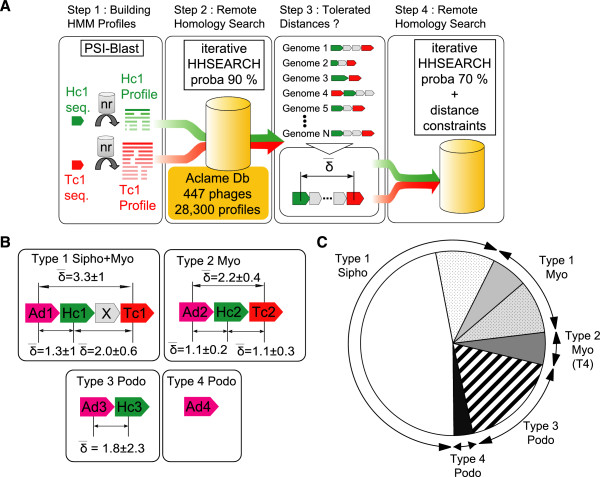
**Identification of head-to-tail connection proteins in tailed bacteriophages and representativeness of the different phage Types. (A)** Schematic representation of the bioinformatics pipeline used to identify remote homologs of head-to-tail connection proteins. Reference HMM profiles of known head-to-tail connection proteins (Table 1), as well as HMM profiles of each of the 28300 protein sequences contained in the Aclame database, were calculated using PSI-Blast (Step 1). These profiles were compared using HHsearch with a stringent probability threshold of 90%, and proteins detected as related to the reference head-to-tail connection proteins were iteratively used as probes in order to detect further homologs (Step 2). Inter-genes distances were then learned (Step 3) and applied as constraints to faithfully retrieve more remote homologs detected at a lower probability threshold (70%) (Step 4). **(B)** Components of the four neck Types are represented using the color code defined in Figure 1. Their mean inter-genes distances and standard errors were calculated as illustrated in Step 3 of panel A. **(C)** Quantitative distribution of the tailed bacteriophages of known morphology and recognized neck Type. Sector color code is the following: white, *Siphoviridae* of Type 1; light gray, *Myoviridae* of Type 1; dark gray, *Myoviridae* of Type 2; hatched, *Podoviridae* of Type 3; black, *Podoviridae* of Type 4. Dotted surfaces in Type 1 sectors correspond to phages with incomplete necks, for which one to two canonical components of the neck were not identified.

A bioinformatics procedure was designed in order to systematically detect the phage head-completion and tail-completion proteins, also called head-to-tail connection proteins, within the Aclame database (Figure 2A). To do so, the characterized head-to-tail connection proteins of phages SPP1, λ, HK97, T4, P22 and Φ29 were taken as starting point. Next, their profiles were compared to the database of 28300 HMMs profiles using the profile-profile comparison algorithm HHsearch [32].

#### Type 1

First, all possible head-to-tail connection proteins Ad1, Hc1 and Tc1 were identified among the 447 genomes by searching for homologs of the SPP1 gp15, gp16 and gp17 proteins using the program HHsearch with a high confidence probability threshold of 90%. Next, the inter-gene distances between the detected Ad1, Hc1 and Tc1 proteins were calculated and averaged on all genomes. The resulting mean inter-gene distances were 1, 2 and 3 for the (Ad1, Hc1), (Hc1, Tc1) and (Ad1, Tc1) couples, respectively, corresponding to the gene ordering: Ad1-Hc1-x-Tc1. They could be organised into a mean distance matrix characteristic of Type 1 neck gene organisation. We also calculated for each couple the standard deviation on the inter-gene distances. From these calculations we deduced a tolerated distance matrix, formed by adding to each mean distance matrix term twice the inter-gene distance standard deviation. The tolerated distance matrix terms were 2, 4 and 5 for the (Ad1, Hc1), (Hc1, Tc1) and (Ad1, Tc1) couples, respectively. Finally, we repeated the iterative profile-profile search with HHsearch, relaxing the probability threshold from 90% to 70%, and keeping only the detected proteins whose gene position was consistent with the tolerated matrix distance. As a result, more than 500 neck proteins were recovered (Table 2).

To control that no false positives were recovered by our relaxed conditions, we checked that we never detected more than one Ad1, one Hc1 or one Tc1 per phage. We also verified our results in the light of well characterized phages. Tc1 being a tail completion protein involved in long tail assembly [26, 48], we anticipated that a neck containing Tc1 would not be found in *Podoviridae* phages. Consistently, no neck containing Tc1 proteins could be detected in *Podoviridae* phages. Some phages do not encode the whole Ad1, Hc1, Tc1 panoply. For instance, the neck of phage λ is made of an Hc1 (called gpFII), a Tc1 (called gpU) but no Ad1. Instead, the gpW protein, with no sequence or structural similarity to Ad1, is required for stabilization of the DNA within the head and for addition of the Hc1 protein. This typical case of functional replacement suggests that GpW is positioned in the virion between the portal and the Hc1 proteins [49]. In our study, a small group of 10 *Siphoviridae* and *Myoviridae* phages exhibit Hc1 and Tc1 proteins but no Ad1 proteins. Within these phages, 4 *Siphoviridae* (in which phage λ) and 1 *Myoviridae* encode a protein similar to gpW using a confidence threshold of 95% and systematically located at distances of 6 to 7 from Hc1 and 9 from Tc1. Thus, our procedure did not detect any spurious remote homologs in the particular cases of necks comprising a gpW-like protein.

We specifically analyzed the genomes lacking only one of the Ad1, Hc1 and Tc1 elements. Searching within the limits imposed by the tolerated distance matrix, 3 Ad1, 5 Hc1 and 13 Tc1 proteins were identified in *Siphoviridae*. These proteins were predicted as having the same secondary structure patterns as the missing ones and were located at distances from the other identified neck proteins compatible with the tolerated distance matrix. They were not automatically selected by our initial profile-profile procedure because their HMM profile was built from a too limited number of sequences (less than 5 homologous sequences in the nr database retrieved at the PSI-Blast stage). Similarly, we identified 1 Ad1 and 1 Tc1 protein in *Myoviridae* phages exhibiting less than 4 homologous sequences in their HMM profile. Finally, 8 additional Ad1 were identified in *Myoviridae* phages exhibiting a Tc1 protein, which are separated by 6 to 8 proteins from Tc1 (the tolerated matrix imposed a maximal value of 5). In the latter case, the 8 Ad1 belong to an Aclame family also comprising 6 other proteins previously identified as Ad1. All these neck proteins are described in Table 2 and Additional file 2: Table S1.

#### Type 2

The head-to-tail connection proteins Ad2, Hc2 and Tc2 were identified by searching for homologs of the T4 gp13, gp14 and gp15 proteins using the program HHsearch with a 90% confidence threshold. Inter-gene distances were calculated between the identified proteins resulting in mean distances of 1, 1 and 2 for the (Ad2, Hc2), (Hc2, Tc2) and (Ad2, Tc2) couples, respectively. This corresponds to the ordering: Ad2-Hc2-Tc2. The tolerated distance was calculated as above by summing the mean and twice the standard deviation inter-gene distances. The tolerated distances were 2, 2 and 3 for the (Ad2, Hc2), (Hc2, Tc2) and (Ad2, Tc2) couples, respectively. As for Type 1, we constrained the search using this inter-gene tolerated distance matrix and repeated the iterative profile-profile search with HHsearch, relaxing the probability threshold from 90% to 70%. As a result, we identified 49 Type 2 neck proteins (Table 2; Additional file 2: Table S1).

#### Type 3

The head-to-tail connection proteins Ad3 and Hc3 were identified by searching for homologs of the P22 gp4 and gp10 proteins with HHsearch and a 90% confidence threshold. A tolerated inter-gene distance of 2 was calculated and used to constrain the search while relaxing the probability threshold of HHsearch at 70%. 89 additional Type 3 neck proteins were thus detected.

We searched for missing Ad3 and Hc3 proteins using the secondary structure predictions and the knowledge of their relative positions in the phage genomes. We identified 1 additional Ad3 protein with consistent predicted secondary structure pattern and inter-gene distance to its Hc3 neighbour. Here again lack of homologous sequences in the original profile hindered the homology detection by the HHsearch algorithm. Moreover, in 3 additional *Podoviridae,* Ad3 and Hc3 were identified but their relative distance was comprised between 3 and 6. Finally, in 8 additional phages (4 *Podoviridae* and 4 unclassified phages), 2 Ad3 and 1 Hc3 were identified per phage, the 2 Ad3 having a relative distance of 1 to 2 and Hc3 having a relative distance to Ad3 of 4 to 15. All these neck proteins are described in Table 2 and Additional file 2: Table S1.

#### Type 4

For this last type, Ad4 proteins were searched using the Φ29 gp11 protein as a seed for the iterative profile-profile procedure. Ten Ad4 proteins were detected, that all belong to *Podoviridae* (Table 2).

### Scoring of the evolutionary divergence between head-neck-tail modules for phage classification

Our analysis identified a set of 9 classes of proteins of the head-neck-tail module comprising 2 classes from the head (major capsid, terminase), 5 from the neck (Portal, Ad, Hc, Tc, Ne) and 2 from the tail (major tail, Sheath). Every class encompasses a number of Aclame protein families that were previously thought unrelated and which are now connected through remote homology relationships by the procedure described above. Taking advantage of this global view of the head-neck-tail organisation, we searched for a metric which would account for the evolutionary divergence between the phage components of the head-neck-tail module. We used a score depending both on the HHsearch probability (ProbaHHsearch) but also on the sequence identity (PercentIdentity) for closely related sequences.
1

The weight of 0.1 assigned to the PercentIdentity factor ensured that the latter term only contributes when ProbaHHsearch caps at 100% because values of ProbaHHsearch below 100% generally correspond to remote homology relationships with PercentIdentity below 25%. For every pair of phages and for all the components of the capsid-neck-tail module (out of the nine possible components), a similarity score was calculated using equation 1. The mean value of all these scores was computed over all the components of the module common to both phages (Portal, Ad, Hc etc.…) in order to yield the similarity score between the pair of phages. It was calculated by adding scores corresponding to superfamilies detected in both phages and ignored superfamilies only detected in one of them.

The mean value was used as a metric of similarity of the capsid-neck-tail module between two phages and a NxN matrix of averaged scores was built from the systematic cross-comparison of all N phages of each type. To group together phages sharing similar average scores, the NxN matrices were then clustered using a hierarchical agglomerative clustering with WPGMA method (Weighted Pair Group Method with Averaging) together with Euclidian distance and a tree corresponding to each neck type was built from these calculations using the ete2 library [50].

For the phage classification step, we took into account all phages with at least two detected head-to-tail connection proteins in the case of Type 1 and 2 phages (including T5 which has only Ad1 and Tc1 proteins), and one detected protein in the case of Type 3 and 4 phages (including N4 which has only one Ad3 protein). In the Webserver, in order to increase sensitivity, all head-to-tail connection proteins detected with a HHsearch threshold higher than 70% are displayed.

### Automatic identification of neck proteins and classification of necks in tailed bacteriophages through the Virfam server

To enlarge our study to newly sequenced phages, we built the Virfam webserver interface through which users can (1) detect the proteins involved in the neck structure of their phage of interest, (2) identify the type of neck, and deduce the likely morphological family of the phage, (3) display the organisation of the genes encoding the major capsid protein, large terminase, portal, neck, major tail and sheath proteins and (4) locate the given phage within the classification built in this work. After submission through this interface, each input protein is aligned using an HHsearch-based protocol against the Virfam collection of 28000 profiles generated for the present work. Phage neck proteins are thus automatically identified and the phage neck architecture is described.

## Results

### Detection of head and tail proteins within the learning dataset

Our structural classification of phages relies on the detection of 9 genes coding for head, neck and tail proteins. We first searched for homologs of 5 head and tail proteins using the homology detection tool HHsearch [32], which relies on the comparison between pairs of multiple sequence alignments (also called profiles). This tool can reveal more remote homologies between proteins than the profile-sequence comparison tool PSI-Blast [31]. It was used with a high confidence probability threshold of 90% to search for the portal, large terminase subunit (noted here TermL but also often abbreviated as TerL) and major capsid protein (MCP) on the head side, the major tail (MTP) and sheath proteins on the tail side, among a set of 447 complete phage genomes, including 303 tailed phages (*Caudovirales*), 119 non tailed phages and 25 uncharacterised phages (taken from Aclame version 0.4; see Virfam server for details). The 5 head and tail proteins could be detected in most tailed phages. Details about the initial sequences used for the search, corresponding to proteins of known 3D structures, are given in the Experimental procedure section and the complete list of detected proteins can be found in Additional file 2: Table S1.

### Detection and classification of head- and tail-completion proteins within the learning dataset

Once the head and tail proteins were identified, we focused on the head-to-tail connection proteins to capture the diversity of neck organisations in tailed phages. We concentrated on the identification of a maximum of highly divergent head-to-tail connection proteins. Therefore, we identified from the literature several proteins characterized as head- and tail- completion proteins in a number of model phages (Table 1), and we search for homologs of these proteins within the genomes of the same 447 completely sequenced phages. Our homology search was performed using the homology detection tool HHsearch, which was now used iteratively to detect highly remote homologs (Figure 2A). A stringent confidence probability threshold of 90% was again chosen to identify homologs of our list of starting proteins (Table 1) among the 28300 proteins encoded by the 447 phages. We detected 633 phage head- and tail-completion proteins (compared to only 282 when using PSI-Blast, see Table 2). Most of them were highly divergent from one another (pairwise identity in the 10-20% range). However, despite this drastic divergence, the 633 detected proteins combined into a limited number of 4 major neck architectures, or neck “Types” (Tables 1 and 3).

**Table 3:** **Detected neck modules in the phages classified in Aclame**

	Type 1 with 4 head-to-tail connection proteins (Ad1, Hc1, Tc1, Ne1)	Type 1 with 2 or 3 head-to-tail connection proteins (and only 1 in brackets)	Type 2 with 3 head-to-tail connection proteins (and only 1 in brackets)	Type 3 with 2 head-to-tail connection proteins (and only 1 in brackets)	Type 4 with an Ad4 head-to-tail connection protein	Number of classified phages	Percentage in Aclame
***Siphoviridae***	126	30				156/169	92
***Myoviridae***	17	21(+7)	16(+1)			62/72	86
***Podoviridae***				44(+3)	10	57/58	98
**Unassigned**	15	3		6	0	24/29	83
Total	158	61	17	53	10	299/328	91

The 299 phages in which neck proteins could be detected are detailed. The distribution of the neck proteins in each phage was interpreted in order to assign a neck architecture (or Type) to the phage. From the neck architecture and the presence or absence of a sheath protein, the morphology of the phage (*Siphoviridae*, *Myoviridae* or *Podoviridae*) could be deduced. For most phages, the morphology obtained from our analysis fits with that proposed by the NCBI (the 4 exceptions are discussed in the text and considered in this table as unassigned by NCBI). To highlight the consistency between our classification and that of the NCBI, we present in the following table the number of Aclame phages as a function of their proposed neck architecture (columns) and NCBI morphological class (lines). More details about the proteins detected for each phage can be found in the Additional file 2: Table S1 and on the Virfam webserver.

These neck Types display different complexities: they involve one to three head-to-tail connection proteins (a fourth protein called Ne1 will be taken into account later on). Type 1 necks (or SPP1-like) are found in *Siphoviridae* and *Myoviridae*. They adopt a structural organisation similar to that of the *Siphoviridae* phage SPP1 and comprise three head-to-tail connection proteins: two head-completion proteins homologous to SPP1 gp15 (Adaptor, noted Ad1) and SPP1 gp16 (Head closure, noted Hc1) and a tail-completion protein homologous to SPP1 gp17 (noted Tc1). Exception to this rule is found for 5 phages (among 219 Type 1 neck phages), which exhibit an adaptor homologous to gpW of phage λ, a protein with a structure distinct from that of SPP1 gp15 [51]. For these 5 phages, the difference is limited to the adaptor since *bona fide* homologs of Hc1 and Tc1 are detected. Type 2 necks (T4-like) are only found in *Myoviridae*. They comprise two head-completion proteins homologous to T4 gp13 (noted Ad2) and T4 gp14 (noted Hc2), and a tail-completion protein homologous to T4 gp15 (noted Tc2). Finally, Type 3 (P22-like), and Type 4 (Φ29-like) necks are mostly found in *Podoviridae*. Type 3 necks have only two head-completion proteins, homologous to P22 gp4 (noted Ad3) and gp10 (noted Hc3), and Type 4 necks have just one connection protein, homologous to Φ29 lower collar/tail tube protein gp11 (noted Ad4) [52].

### Using the gene context to detect additional head- and tail-completion proteins within the neck module

At this stage, we observed that several phages could be assigned to one of the four types defined above but lacked one of the neck gene. In particular, about 13, 28 and 24% of Type 1 necks lacked an Ad1, Hc1 and Tc1, respectively, and about 85% of Type 3 necks lacked an Ad3 when using HHsearch with a confidence probability threshold of 90%. We wondered whether absence of these neck proteins was real or due to, once again, the huge sequence divergence.

To further explore the existence of putative very remote homologs, we lowered the confidence probability threshold of HHsearch to 70%. However, such a threshold was found too permissive in some cases and led to the detection of spurious homologs. To improve the detection specificity, we implemented an additional constraint related to the conserved head-to-tail connection module organisation observed among most of the phages (Figure 2A). In practice, the distances between the previously detected head-to-tail connection genes were calculated from their positions in the genomes. Figure 2B summarizes the mean distances (and corresponding standard deviations) observed between Type 1, 2, 3 and 4 detected genes. We observed strong constraints on the relative position of the head-to-tail connection protein genes, from which we defined distance interval thresholds, corresponding to the mean +/- two standard deviations. Using these distance thresholds, we could decrease the HHsearch confidence threshold from 90% down to 70% and detect additional head-to-tail connection proteins without inclusion of spurious homologs.

As presented in Table 2, 10 Ad1, 4 Hc1, 27 Tc1 and 30 Ad3 additional proteins could be identified. In total, 747 head-to-tail connection proteins were found. We detected these proteins in 299 phages, which were unevenly distributed: Type 1 neck phages were the most prevalent (219 phages fall into this large class), while Type 2, 3 and 4 necks were found in 17, 53 and 10 phages, respectively (Figure 2C and Table 3). The specificity of our procedure was controlled by the fact that head-to-tail connection proteins were never identified in phages annotated by the NCBI as non-tailed phages: they were all detected in *Caudovirales* or unclassified phages. Furthermore, as already noticed, the proposed Type classes are consistent with the morphological subfamilies *Siphoviridae*, *Myoviridae* and *Podoviridae.* Two exceptions were identified corresponding to phages that are most probably mis-assigned in the NCBI database as deduced from recent experimental data: (i) 3 phages annotated as *Siphoviridae* for which we detected *Podoviridae*-like Ad3 and Hc3 proteins specific to Type 3 neck; these phages are Stx1 and Stx2 converting phages, which are closely related to 933 W unambiguously recognized as a *Podoviridae*[53, 54]; (ii) 1 phage annotated in NCBI as *Siphoviridae* for which we detected Type 1 neck proteins and a sheath; this phage was recently reported as a *Myoviridae* by [55].

### Detection of a new Ne1 superfamily gene in the neck module of Type 1 phages

**Figure 3:**
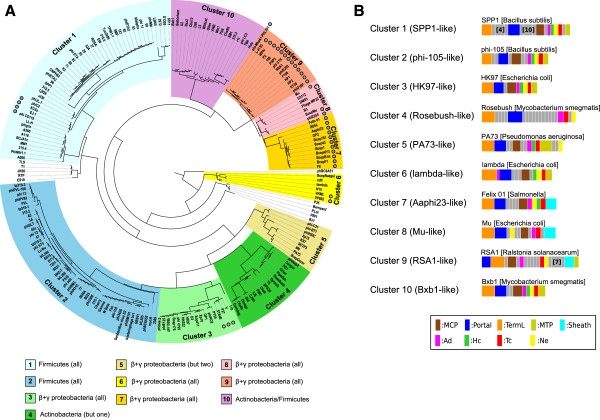
**Classification of the Type 1 bacteriophages. (A)** Tree representation of Type 1 phage similarities, built from a hierarchical agglomerative clustering procedure applied to a matrix of similarity scores between pairs of phages (combining HHsearch probabilities and percentage of identity) and represented using the ETE2 library [50]. The different branches of the tree were sorted into 10 Clusters, highlighted by different background colors. Phage names labelled by black circles filled in grey indicate the *Myoviridae* phages. Bacterial hosts of the phages are indicated in the bottom for each Cluster with the same color code as in the classification tree, in order to highlight the consistency between the Cluster and host phyla. **(B)** Gene organisation of a representative phage of each Cluster. A more complete view of gene organisation sorted by Clusters is presented on the Virfam webserver, in order to highlight the consistency between the Cluster and neck gene order distributions.

We analyzed the genome organisation of the head, neck and tail proteins in the 4 neck Types, and deduced their corresponding average gene organisation (Additional file 1: Figure S1). We further explored whether any unannotated gene superfamily might emerge in the vicinity of the neck genes. Typically, in all Type 1 phages, an unannotated gene encoding a protein homologous to SPP1 gp16.1, designated hereafter *Ne1* (for neck protein of Type 1), was detected between the head and tail genes, most frequently positioned as Ad1-Hc1-Ne1-Tc1 (Ne1 is displayed in yellow in a sample of phage genomes in Figure 3). Ne1 proteins exhibit an amazing versatility of sizes, ranging from 56 to 231 residues, which probably precluded their previous identification as belonging to the same protein superfamily. However, most Ne1 proteins were detected with a HHsearch confidence threshold higher than 95% (Additional file 1: Figure S2). The remarkable systematic presence of their gene in Type 1 neck modules suggests a critical role in the head-to-tail connection assembly or function.

Scattered experimental data exist on the function of Ne1 proteins. In the Siphophage λ, in the absence of the Ne1 protein GpZ, viral particles are produced, but they have low infectivity [56]. GpZ was proposed to be part of the virion tail [57] and to represent a conserved family of tail proteins [58], although the systematic presence of a remote homolog in almost all Type 1 phages remained hidden due to sequence divergence. The phenotype is slightly different in mutants of the Ne1-encoding gene *gpS* of Myophage P2: phages form tails that are unable to attach to heads, and are non-infectious, suggesting a defect in the head-tail joining process [59]. Because of the systematic presence of the Ne1 gene in the neck module and of the few experimental data pointing towards an important role of Ne1 in virion head-to-tail connection assembly and infectivity, we further included the Ne1 gene in our description of Type 1 necks (Figure 3).

### Phage classification from the “head-neck-tail” module analysis

From our analysis of phage necks, we deduced that Type 1 necks are the most prevalent. Considering the large size of this group, we further subdivided it according to a metric which accounts for the “average” evolutionary divergence among the various proteins composing the defined head-neck-tail module. The probability that two protein profiles are similar as calculated by the HHsearch program is an interesting metric for quantifying the degree of remote homology between proteins. However, in case of close homology, typically above 35% of sequence identity, HHsearch probabilities peak at 100% and are not discriminative. To account for the whole spectrum of sequence divergence, we used a similarity score primarily driven by the HHsearch probability but with an additional contribution of sequence identity to properly recognize closely related phages (see Methods). For every pair of phages, similarity scores were calculated between all components of the head-neck-tail module (comprising up to 9 proteins) and then averaged to yield a mean score reflecting the similarity between the head-neck-tail modules of the phage pair. From this metric, a hierarchical agglomerative clustering procedure was used to obtain a tree representation of phage similarities (Figure 3A; this analysis is extended to Types 2–4 phages on the Virfam webserver).

The clustering procedure discriminated ten major Clusters within the Type 1 phage family. Inside each Cluster, HHsearch probabilities between homologous proteins are often close to 100%, but even in these cases, identities between protein sequences can be very low (in the range 10-20%) (analysis of a representative subset of phages of Type 1 Cluster 1 in Additional file 1: Figure S3A; similar analyses are available on other Types and Clusters on the Virfam webserver). Between distinct Clusters, HHsearch mean probabilities are on average below 50%, and the connection between these groups is achieved through a limited number of proteins bridging together the whole ensemble (Additional file 1: Figure S3B and the Virfam webserver, by clicking on the Type or Cluster of interest at http://biodev.cea.fr/virfam/tables_results/help/AllTypes.html).

As illustrated in Figure 3A, the Clusters group together phages that infect hosts from the same phylogenetic clades supporting the consistency of the classification. A characteristic of Type 1 phage is that their hosts spread over the whole bacterial phylogenetic tree which is not the case for all the Types defined in our study (see Discussion). Also, genome organization of the head-neck-tail module is partitioned in a homogeneous manner among the Clusters although no prior constraint related to gene order or to inter-gene distance was included in the clustering metrics (Figure 3B; see also the Virfam webserver). An additional remarkable feature standing out from this classification is that phages from the *Myoviridae* family do not tend to cluster together. Phages with a sheath in their genome are spread over more than half of the Clusters, namely Clusters 1, 3, 6, 7, 8 and 9, together with closely related phages deprived from a contractile system. Such versatility in the presence/absence of a sheath protein had previously been noticed in the case of phages related to Mu (grouped in Cluster 8) [15] and appears as a general property of Type 1 phages.

### Automatic identification of neck proteins and classification of necks in tailed bacteriophages through the Virfam server

**Figure 4:**
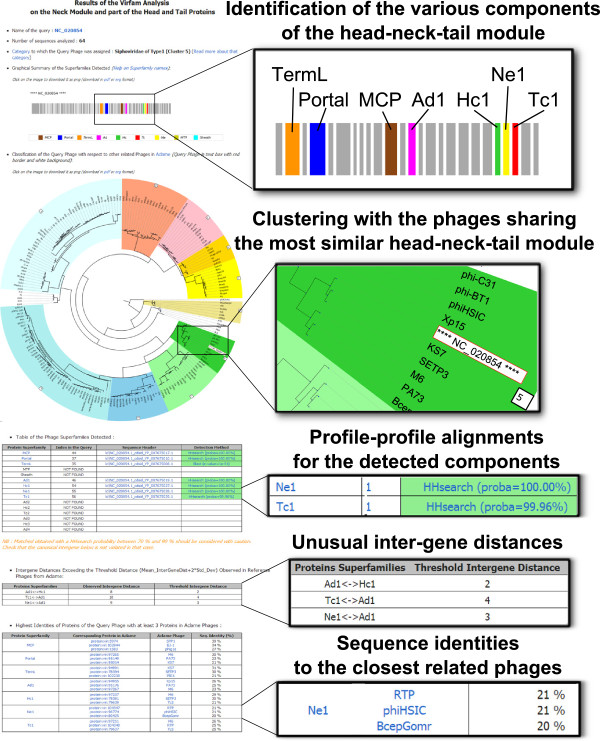
**Typical analysis of a phage that is unclassified in the NCBI database, as provided by the Virfam Webserver.** The Virfam webserver can be used to identify the head-neck-tail module in any bacteriophage genome from the set of ordered protein sequences. In the output, the Type and Cluster inferred from the detected superfamilies will be returned together with **(A)** a graphical representation of the components identified in the genome, **(B)** a clustering of the query phage with respect to those of same Type in the Aclame database, **(C)** a detailed report from the HHsearch analysis providing the corresponding alignments, **(D)** a warning in case unusual inter-gene distances are detected, **(E)** a list of the most similar proteins present in the Aclame database with a connexion to the corresponding phage and its pre-computed head-neck-tail module analysis page.

Our analysis and resulting classification enabled to classify 299 phages within 328 tailed and unclassified Aclame phages. To enlarge our study to newly sequenced phages, we built the Virfam webserver interface (Figure 4). We challenged the robustness and representativeness of our Webserver by investigating 624 new phage genomes available at the NCBI (July 2013 update). Within these phages, 19 are annotated as non-tailed phages, and indeed could not be classified by our approach. The 605 remaining phages are either described as *Caudovirales* (n = 601) or unclassified by the NCBI (n = 4). 93% of these phages could be sorted among the four neck Types by our approach (Table 4). Interestingly, the distribution of the different Types of phages is similar to that observed in the learning phage database. Some phage subfamilies are enriched. For example, at least 7 N4-like phages [60] are now found within Type 3 neck phages, and they all lack Hc3, thus confirming that the head-closure protein of N4-like phages is highly divergent. In the case of Type 1 phages, the distribution within Clusters is also similar to that observed for the learning set: 89% of these phages could be assigned to one of the previously defined Clusters. We conclude that the classification constructed on the 299 Aclame phage genomes is relevant for a global classification of most of the 624 newly analyzed genomes. The Virfam server is thus a robust tool for the classification of novel tailed phages and the identification of their structural organization at the head-to-tail interface.

**Table 4:** **Neck modules detected through the Web server in phages classified in the NCBI database but not in Aclame**

	Type 1	Type 2	Type 3	Type 4	Total	Percentage in NCBI
*Siphoviridae*	278				278 (244 annotated as *Siphoviridae*)	94
*Myoviridae*	81	75			156 (138 annotated as *Myoviridae*)	85
*Podoviridae*			116	10	126 (101 annotated as *Podoviridae*)	99
Total	359	75	116	10	560	93

Percentages are calculated within the *Siphoviridae, Myoviridae* and *Podoviridae* subfamilies described by the NCBI. Total percentage corresponds to the number of assigned phages (560) compared to the total number of *Caudovirales* and unclassified phages of the analyzed NCBI database (624–19 non tailed bacteriophages =605).

## Discussion

Understanding the genomic and structural diversity of phages is a critical challenge, stimulated by the increasing number of available phage sequences. 96% of the bacteriophages isolated so far are tailed bacteriophages. Here, we described the structural diversity of a set of proteins essential for tailed phage virion assembly and function, comprising both the semi-conserved capsid, portal, terminase, major tail and sheaths proteins and the more divergent head-to-tail connection proteins. Neck proteins are particularly important for capsid completion, hence for the phage life cycle: they form a channel between the head and the tail, that has to close rapidly after DNA packaging in order to avoid DNA leakage and to reopen after recognition of the targeted bacteria in order to allow infection. Despite their diversity, they are thus submitted to significant evolutionary constraints which we attempted to reveal by combining profile-profile comparisons with gene context analyses. Our ambition was to evaluate the protein sequence and composition diversity at the head-to-tail connection and to use this knowledge to propose a detailed phage classification related to phage morphology.

### Robustness of the proposed classification

We first successfully detected a large set of structural proteins present in 299 phages, comprising 92% of the *Siphoviridae,* 86% of the *Myoviridae* and 98% of the *Podoviridae* of our learning database (Additional file 2: Table S1). We identified a Type and thus a neck architecture for these phages, and proposed a classification within phages of the same Type and thus with the same neck structure. This approach enabled classification of 91% of the *Caudovirales* and unclassified phages of the learning database and 93% of the *Caudovirales* and unclassified phages of the NCBI database (Additional file 3: Table S2). The higher success of our procedure in the case of the NCBI database is linked to an overrepresentation of Type 2 phages, which are more closely related and thus easier to detect.

Interestingly, we find that the resulting phage classification is correlated to the targeted bacteria phylogenetic tree: if Type 1 phages infect a very large number of hosts spanning the whole bacterial phylogenetic tree and in particular Cyanobacteria, Proteobacteria and Firmicutes, Type 2 and Type 3 are only found so far to infect Cyanobacteria and Proteobacteria and Type 4 are only detected as infecting Firmicutes (Additional file 1: Figure S4).

It was proposed that within the neck, the Portal protein alone is already an efficient marker for phage classification as a function of virion architecture (Comeau et al. [61]). Using our scoring function calculated on the Portal protein alone, we built a tree of all the Aclame phages (Additional file 1: Figure S5A). The resulting Type classification is similar to that described in our study (only two Type 3 phages were not correctly clustered with the remaining Type 3 phages). Using the three MCP, Portal and TermL protein markers did not improve the discrimination of Types (Additional file 1: Figure S5B). We also observed an overall good consistency between the Portal classification and our Type 1 tree generated using multiple markers.

**Figure 5:**
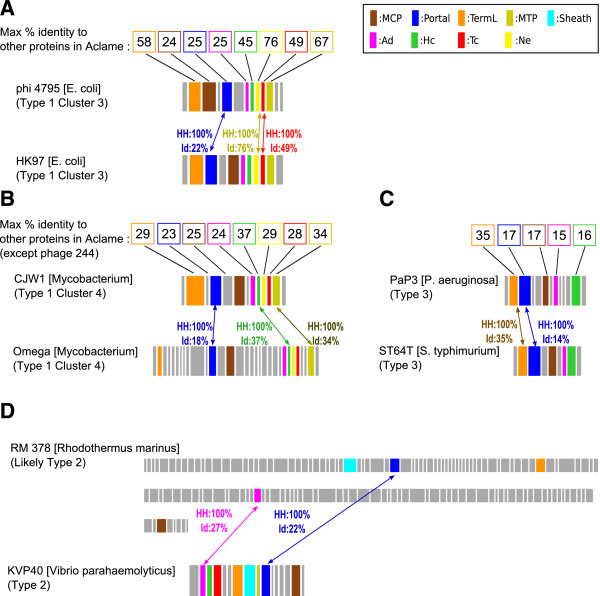
**Interest of using a multi-protein analysis for phage classification when Portal sequence diverged more than other proteins of the head-neck-tail module.** Four pairwise comparisons between the head-neck-tail modules of phages whose Portal proteins have significantly diverged with respect to other proteins of the head-neck-tail module. **(A)** Phage phi 4795, assigned to Type 1 Cluster 3, has a Portal sharing less than 25% identity with any other protein in Aclame database. Its TermL, Ne1, Tc1 and MTP proteins present a significantly higher conservation profile. In particular, compared with HK97, Ne1 and Tc1 share 76% and 49% identity with their homologs in phi 4795, respectively, while Portal only shares 22%. **(B)** Phage CJW1, assigned to Type 1 Cluster 4, has a Portal sharing less than 23% identity with any other protein in Aclame database. Proteins such as Hc1 or MTP share a higher conservation profile. In particular, they share 37% and 34% identity with Ne1 and Tc1 of phage Omega, respectively while the Portal only shares 18%. **(C)** Phage PaP3, assigned to Type 3, has a Portal sharing less than 17% identity with any of the other proteins of Aclame database. In contrast, its TermL diverged to a lesser extent sharing 35% identity with that of phage ST64T. **(D)** RM 378 is among the most divergent phages assigned to Type 2. Only an Ad2 protein could be detected when searching for its head-to-tail connection proteins. Although its Portal shares at most 22% identity with the closest Portal of other phages of Aclame, its Ad2 protein could be recognized with higher identity (27% identity with KVP40) providing stronger support for the assignment of this phage to the Type 2 category.

However, significant discrepancy is observed in the phages hosted by Actinobacteria (mainly Mycobacteria), which were found to group in Clusters 4 and 10 in our Type 1 multi-protein classification. If only the Portal is used, many phages populating Cluster 4 are spread in different branches of the tree irrespective of the nature of their hosts (highlighted by a mark in Additional file 1: Figure S5A). Moreover, phages Giles and Min1 that were connected to Cluster 10 are not anymore clustered with phages from Actinobacteria. Therefore, observations on highly divergent phages from *Podoviridae* or from Clusters 4 and 10 show that the strategy we propose, using neck proteins, is able to discriminate phages hosted by bacteria from the same Phylum. The issue noticed for members of Clusters 4 and 10 can also be perceived for Cluster 3 (hosted by Proteobacteria) which tends to mix within cluster 2 (hosted by Firmicutes) in the Portal-only tree (Additional file 1: Figure S5A). Altogether our results show that the Portal protein may diverge in evolution while head-to-tail connection proteins still show consistent evolutionary links with proteins from other phages (Figure 5). The metric used to establish the phage similarity matrix account for that property. The fact that our multi-protein classification in four Types is (i) globally consistent with Portal-only classification (ii) successful in difficult cases in recognizing phages hosted in Bacteria of the same phylum supports the idea that neck proteins bring useful signal to the classification of phages.

### Comparison to previously described whole genome classifications

Previous studies have shown that *Podoviridae* and *Myoviridae* could be classified using BLAST-based tools and phage distance calculations based on global shared genes [9, 14, 61]. These phages were divided into subfamilies, which were recognized as biologically significant and are available on the ICTV website [62]. *Podoviridae* were classified into two main subfamilies, *Picovirinae* (comprising Φ29-like phages and several other genera) that corresponds to the present Type 4, and *Autographivirinae* (comprising T7-like phages and several other genera) that perfectly matches with one branch of our Type 3 (Additional file 1: Figure S6). Other groups of the Type 3 classification are close to the tentative P22-containing subfamily and to several of the unclassified genera (Additional file 1: Figure S3). Hence, our analysis connects in a hierarchical and consistent manner several independent groups of *Podoviridae*, such as *Autographivirinae* and P22-like phages, on the basis of the similarities between their head-to-tail modules.

The ICTV classification of *Myoviridae* is more difficult to overlap with ours. These phages were previously classified into three main subfamilies, *Peduovirinae* (comprising P2-like and HP1-like phages)*, Teequatrovirinae* (comprising T4-like phages and several other genera) and *Spounavirinae* (comprising SPO1-like and Twort-like phages) [9, 61]*,* plus several other independent genera. The *Teequatrovirinae* subfamily corresponds to the Type 2 category of our classification*.* But in our Type 1 class, *Myoviridae* from the other subfamilies or genera are often mixed with *Siphoviridae* in Type 1 Clusters, suggesting that their contractile character has been acquired or lost in a versatile manner throughout phage evolution. Consistent clusters were however retrieved within these myophages. P2-like, Mu-like and AaΦ23-like phages that were described as very different, based on BLAST- analyses [9, 61] are well partitioned into different Type 1 clusters. *Peduovirinae* perfectly matches Type 1 Cluster 9*,* while four previously independent genera of *Myoviridae* are now grouped into Type 1 Cluster 7. Finally, the *Spounavirinae* subfamily is left as a special category in which only one Ad1 component could be identified but no Hc or Tc subunits recognized so far. Although recognized as Type 1-like, they could not be further clustered and will be interesting to investigate further.

Altogether, our automatic classification tool consistently retrieved the known phage subfamilies as they were described for *Podoviridae* and *Myoviridae*. The remote homology analysis further provides evidence on how to connect independent subgroups based on their neck architecture similarities. Moreover, it supports a classification for the *Siphoviridae* and highlights the neck structural relationships between *Sipho-* and *Myoviridae.*

### A common structural core for all neck Types?

Our classification highlights the representativeness of four different Types of neck architecture. Each Type is characterized by a set of specific head-to-tail connection proteins. It is also characterized by a specific distribution of phage genome sizes. Tiny phages (encoded by less than 30 genes) generally exhibit Type 4 necks, common size phages (encoded by 31 to 150 genes) present either Type 1 or Type 3 necks, and large phages often show Type 2 necks (Additional file 1: Figure S7 and Additional file 1: Text S1). Physical explanations might be discovered in the future that explain this correlation between neck proteins and genome size. Interestingly, our scoring of phage protein homologies also suggests that the different neck Types might share some common structural properties. Indeed, structural analogies exist within each category of neck components, the so-called Ad, Hc and Tc. For instance all four Ad1, Ad2, Ad3 and Ad4 exhibit 4 to 5 predicted α-helices and HHsearch confidently predicts homology between an Ad1 and an Ad3 (Additional file 1: Text S2). Comparison between Ad1 and Ad3 protein structures consistently revealed that they share the same α-helical bundle fold (gp15 of SPP1 [63], gp6 of HK97 [64] for Ad1 and gp4 of P22 for Ad3 [65]). Similar observations were made on the Hc1, Hc2 and Hc3 proteins: they are all predicted to fold into a β - strand rich structure and HHsearch suggests homology between a Hc1 and a Hc2 (Additional file 1: Text S2). As regards Tc1 and Tc2, secondary structure prediction and our HHsearch calculations again predict that they share a common structural core. Moreover, recent determination of the three-dimensional structure of a Tc2 protein (gp15 from phage T4) uncovers its structural analogy with a Tc1 protein, gpU from phage λ Fokine et al. [66]. Altogether, these observations support the existence of structural similarities between the components of the various neck types classified in this work and advocate for the existence of a common, highly remote, virion ancestor showing neck structural characteristics close to those highlighted in this study. Given the huge diversity of Type 1 phages highlighted in this work, their ability to populate all bacterial phyla and some archaeal ones (data not shown), these phages are well suited to represent the ancestral neck organization in *Caudovirales*.

## Conclusions

Through the Virfam webserver, it is now possible to detect both the relatively well-conserved capsid, large terminase and tail genes and the highly diverse head-to-tail connection genes. Differences at the head-to-tail connection in protein number and fold were highlighted within tailed phages, and large differences were observed between phage subfamilies (*Siphoviridae, Myoviridae, Podoviridae*). We showed that these proteins are crucial to define the type of morphology a phage likely adopts. We reasoned that going further into the description of the phage head-to-tail differences should provide a classification based on detailed virion morphology. Our study demonstrated that a combination of sequence and profile-profile comparison scores can be used to delineate consistent subgroups in the remote homology space of *Caudovirales* virions. Given the high detection rate of the neck genes provided by Virfam, most new phages can now be positioned within a “head-neck-tail module based” classification. Outside this specific module it is clear that each phage has also evolved and adapted to its specific environment. Versatility and reshuffling of gene functions in phage genomes, which often confuse a phylogenetic classification of phages, may have been less intense in the head-neck-tail module. This module may thus serve as a probe for further exploring the evolution of phages.

Our methodology enables classification and comparison of viromes. Terminases and major tail proteins were previously proposed as phage markers. Unlike these single gene approaches, our characterisation of the phage head-neck-tail module is not hindered when one of the genes is particularly divergent, and this gene can even be detected, provided its genome position is canonical. Identification of neck genes within a phage genome further suggests that this genome codes for a functional virion, thus differentiating phages and other mobile genetic elements. Altogether, as the Virfam server allows for an automatic classification of phages, it should facilitate assignment of viromes and detection of functional phages from bacterial metagenomic samples.

## Availability and requirements

The Virfam webserver is accessible at the following address: http://biodev.extra.cea.fr/virfam/. The input is a phage genome. The order of the genes must be preserved because it is used in our analysis. Further information on the input format is available by clicking on the “how to proceed” button of the main page.

## Electronic supplementary material

Additional file 1: Figure S1: Mean arrangement of the neck genes relatively to the head and tail genes in Type 1 *Siphoviridae*, Type 1 *Myoviridae*, Type 2 *Myoviridae*, Type 3 *Podoviridae* and Type 4 *Podoviridae*: (A) Inter-genedistance matrices resulting from the analysis of the Aclame genomes, (B) Graphical representation ofthe average gene organization for each neck Type. **Figure S2.** Secondary structure analysis of Type 1 neck Ne1 proteins identified in (A) *Siphoviridae* and (B) *Myoviridae.* **Figure S3.** Matrices of profile-profile comparison and identity scores (A) within a typical Type 1 Cluster 1 and (B) between Type 1 Clusters. **Figure S4.** Relationship between the identified phage neck Types and the host bacteria phyla. **Figure S5.** Global tree calculated using only (A) the Portal or (B) the MCP-TermL-Portal triad to compute the similarity matrix between phages. **Figure S6.** Tree representation of the clustering applied to bacteriophages belonging to the Type2, Type3 and Type4. **Figure S7.** Distribution of the Aclame phages of known morphology as a function of their genome size and neck Type. **Text S1.** Relationship between neck structural organisation and genome size in tailed bacteriophages. **Text S2.** Structural analogies between the different neck Types. **Method S1.** Procedure to assess the sensitivity of PSI-Blast search for Table 2. **Tables S3.** Profile identifiers used to test the procedure of remote homology detection using PSI-Blast. (PDF 1 MB)

Additional file 2: Table S1: Table of the 447 phages from Aclame with all proteins assigned by the virfam strategy. (XLSX 111 KB)

Additional file 3: Table S2: Table of the 623 most recent phages from the NCBI with all proteins assigned by the virfam strategy. (XLSX 174 KB)
