# Supplementary material for: Automated classification of tailed bacteriophages according to their neck organization
Source: BMC Genomics. 2014 Nov 27;15(1):1027. doi: 10.1186/1471-2164-15-1027 (PMC4362835; doi:10.1186/1471-2164-15-1027)
Supplement: Supplementary file 1 — Additional file 1: Figure S1: Mean arrangement of the neck genes relatively to the head and tail genes in Type 1 Siphoviridae, Type 1 Myoviridae, Type 2 Myoviridae, Type 3 Podoviridae and Type 4 Podoviridae: (A) Inter-genedistance matrices resulting from the analysis of the Aclame genomes, (B) Graphical representation ofthe average gene organization for each neck Type. Figure S2. Secondary structure analysis of Type 1 neck Ne1 proteins identified in (A) Siphoviridae and (B) Myoviridae. Figure S3. Matrices of profile-profile comparison and identity scores (A) within a typical Type 1 Cluster 1 and (B) between Type 1 Clusters. Figure S4. Relationship between the identified phage neck Types and the host bacteria phyla. Figure S5. Global tree calculated using only (A) the Portal or (B) the MCP-TermL-Portal triad to compute the similarity matrix between phages. Figure S6. Tree representation of the clustering applied to bacteriophages belonging to the Type2, Type3 and Type4. Figure S7. Distribution of the Aclame phages of known morphology as a function of their genome size and neck Type. Text S1. Relationship between neck structural organisation and genome size in tailed bacteriophages. Text S2. Structural analogies between the different neck Types. Method S1. Procedure to assess the sensitivity of PSI-Blast search for Table 2. Tables S3. Profile identifiers used to test the procedure of remote homology detection using PSI-Blast. (PDF 1 MB) [file 12864_2014_6841_MOESM1_ESM.pdf]

# Supplementary materials

## Supplementary figures

1 – Mean arrangement of the neck genes relatively to the head and tail genes in Type 1 *Siphoviridae*, Type 1 *Myoviridae*, Type 2 *Myoviridae*, Type 3 *Podoviridae* and Type 4 *Podoviridae*: (A) Inter-gene distance matrices resulting from the analysis of the Aclame genomes, (B) Graphical representation of the average gene organization for each neck Type.

2- Secondary structure analysis of Type 1 neck Ne1 proteins identified in (A) *Siphoviridae* and (B) *Myoviridae.*

3 – Matrices of profile-profile comparison and identity scores (A) within a typical Type 1 Cluster 1 and (B) between Type 1 Clusters.

4 – Relationship between the identified phage neck Types and the host bacteria phyla.

5 – Global tree calculated using only (A) the Portal or (B) the MCP-TermL-Portal triad to compute the similarity matrix between phages.

6 – Tree representation of the clustering applied to bacteriophages belonging to the Type2, Type3 and Type4.

7 - Distribution of the Aclame phages of known morphology as a function of their genome size and neck Type.

## Supplementary texts

1 - Relationship between neck structural organisation and genome size in tailed bacteriophages

2 - Structural analogies between the different neck Types

## Supplementary methods

1 – Procedure to assess the sensitivity of PSI-Blast search for Table 2

## Supplementary tables

1 - Table of the 447 phages from Aclame with all proteins assigned by the virfam strategy.

2 - Table of the 623 most recent phages from the NCBI with all proteins assigned by the virfam strategy.

3 – Profile identifiers used to test the procedure of remote homology detection using PSI-Blast

## Supplementary figure 1

## A. Inter-gene distance matrices resulting from the analysis of the Aclame genomes

Type 1 *Siphoviridae*

Mean Distance Matrix over 156 mges

```
   MCP PORTAL TERML   MTP   Ad1   Hc1   Ne1   Tc1
[[  0.    3.3   4.6   6.    2.    3.    4.1   4.9]
 [  0.    0.    1.6   9.4   5.6   6.3   7.8   8.6]
 [  0.    0.    0.   10.7   7.1   7.6   9.4  10.2]
 [  0.    0.    0.    0.    4.3   3.2   2.1   1.1]
 [  0.    0.    0.    0.    0.    1.2   2.4   3.1]
 [  0.    0.    0.    0.    0.    0.    1.2   2.1]
 [  0.    0.    0.    0.    0.    0.    0.    1.2]
 [  0.    0.    0.    0.    0.    0.    0.    0. ]]
STD Error Distance Matrix
[[ 0.   1.9  2.5  2.6  1.4  1.9  2.2  2.2]
 [ 0.   0.   1.9  4.   3.1  3.6  3.5  3.2]
 [ 0.   0.   0.   4.4  4.4  4.2  4.7  4.2]
 [ 0.   0.   0.   0.   1.8  1.6  0.8  0.5]
 [ 0.   0.   0.   0.   0.   1.1  1.3  1.4]
 [ 0.   0.   0.   0.   0.   0.   0.9  1.1]
 [ 0.   0.   0.   0.   0.   0.   0.   0.6]
 [ 0.   0.   0.   0.   0.   0.   0.   0. ]]
```

Subfamilies of genes whose relative positions are tightly restrained (STD Error Distance < 3, underlined in yellow in the matrices):

- MCP, TermL, Portal
- MCP, Ad1, Hc1, Ne1, Tc1, MTP

Type 3 *Podoviridae*

Mean Distance Matrix over 47 mges

```
   MCP PORTAL TERML   Ad3   Hc3
[[  0.    2.8   7.6   2.4   4. ]
 [  0.    0.    6.7   5.2   6.9]
 [  0.    0.    0.    9.2  10. ]
 [  0.    0.    0.    0.    1.8]
 [  0.    0.    0.    0.    0. ]]
STD Error Distance Matrix
[[ 0.   1.1  4.8  1.9  3.6]
 [ 0.   0.   6.9  2.6  4.3]
 [ 0.   0.   0.   3.8  4.7]
 [ 0.   0.   0.   0.   2.2]
 [ 0.   0.   0.   0.   0. ]]
```

Subfamilies of genes whose relative positions are tightly restrained (STD Error Distance < 3, underlined in yellow in the matrices):

- MCP, Portal, Ad3
- Ad3, Hc3

Type 1 *Myoviridae*

Mean Distance Matrix over 45 mges

```
   MCP PORTAL TERML   MTP Sheath   Ad1   Hc1   Ne1   Tc1
[[  0.    4.1   4.9  10.2   9.3   2.1   3.7   5.1   5.4]
```

```
 [  0.    0.    1.8   14.   13.3   6.1   8.3   8.7   9.5]
 [  0.    0.    0.    15.   14.3   6.9  10.6   9.5  10.3]
 [  0.    0.    0.    0.    1.    8.4   4.5   5.6   4.9]
 [  0.    0.    0.    0.    0.    7.4   3.5   4.7   3.8]
 [  0.    0.    0.    0.    0.    0.    1.8   3.5   3.5]
 [  0.    0.    0.    0.    0.    0.    0.    1.2   1.5]
 [  0.    0.    0.    0.    0.    0.    0.    0.    1.6]
 [  0.    0.    0.    0.    0.    0.    0.    0.    0. ]]
STD Error Distance Matrix
[[  0.    1.6   3.4   5.9   5.9   0.9   3.9   2.7   2.2]
 [  0.    0.    2.1   6.    6.    2.4   8.4   3.6   2.9]
 [  0.    0.    0.    5.7   5.6   3.9  10.9   3.9   3.5]
 [  0.    0.    0.    0.    0.2   6.3   5.7   3.6   4. ]
 [  0.    0.    0.    0.    0.    6.2   4.7   3.5   3.9]
 [  0.    0.    0.    0.    0.    0.    2.1   2.8   2.3]
 [  0.    0.    0.    0.    0.    0.    0.    1.2   1.6]
 [  0.    0.    0.    0.    0.    0.    0.    0.    1.4]
 [  0.    0.    0.    0.    0.    0.    0.    0.    0. ]]
```

Subfamilies of genes whose relative positions are tightly restrained (STD Error Distance < 3, underlined in yellow in the matrices):

- TermL, Portal
- MTP, Sheath
- MCP, Portal, Ad1, Tc1
- Ad1, Hc1, Ne1, Tc1

<u>Type 2 *Myoviridae*</u>

Mean Distance Matrix over 17 mges

```
   MCP  PORTAL TERML   MTP  Sheath  Ad2   Hc2   Tc2
[[  0.    4.8   8.4   5.8   7.2  14.2  13.2  12.1]
 [  0.    0.    3.6   1.    2.4   9.4   8.4   7.2]
 [  0.    0.    0.    2.6   1.2   5.9   4.8   3.7]
 [  0.    0.    0.    0.    1.4   8.4   7.4   6.2]
 [  0.    0.    0.    0.    0.    7.    5.9   4.8]
 [  0.    0.    0.    0.    0.    0.    1.1   2.2]
 [  0.    0.    0.    0.    0.    0.    0.    1.1]
 [  0.    0.    0.    0.    0.    0.    0.    0. ]]
STD Error Distance Matrix
[[ 0.   0.5  1.4  0.5  1.6  3.7  3.7  3.8]
 [ 0.   0.   1.4  0.   1.7  4.   4.   4.1]
 [ 0.   0.   0.   1.4  0.8  3.1  3.1  3.2]
 [ 0.   0.   0.   0.   1.7  4.   4.   4.1]
 [ 0.   0.   0.   0.   0.   2.8  2.8  2.9]
 [ 0.   0.   0.   0.   0.   0.   0.2  0.4]
 [ 0.   0.   0.   0.   0.   0.   0.   0.3]
 [ 0.   0.   0.   0.   0.   0.   0.   0. ]]
```

Subfamilies of genes whose relative positions are tightly restrained (STD Error Distance < 3, underlined in yellow in the matrices):

- MCP, Portal, TermL, MTP, Sheath
- Ad2, Hc2, Tc2, Sheath

<u>Type 4 *Podoviridae*</u>

Mean Distance Matrix over 10 mges

```
  MCP  PORTAL Ad4
[[ 0.   2.2   4.3]
 [ 0.   0.   1. ]
```

```
 [ 0.    0.    0. ]]
STD Error Distance Matrix
[[ 0.    2.3   4.4]
 [ 0.    0.    0. ]
 [ 0.    0.    0. ]]
```

Subfamilies of genes whose relative positions are tightly restrained (STD Error Distance < 3, underlined in yellow in the matrices):

- MCP, Portal
- Portal, Ad4

## B. Graphical representation of the average neck gene organization in phages with known morphology and neck Type

The numbers of identified phages with the corresponding gene arrangement are indicated in brackets. 248 Type 1 and 3 phages share a similar organization, defined by the cluster TermL-(x)$_{1-3}$-Portal-(x)$_{2-3}$-MCP-x-Ad-(x)$_{0-1}$-Hc. In the remaining 17 *Myoviridae* of Type 2 and 10 *Podoviridae* of Type 4, specific conserved patterns are observed. The gene names and colors are defined in Figure 1. Ne1 corresponds to an additional Type 1 neck protein identified through this work (see Results section).

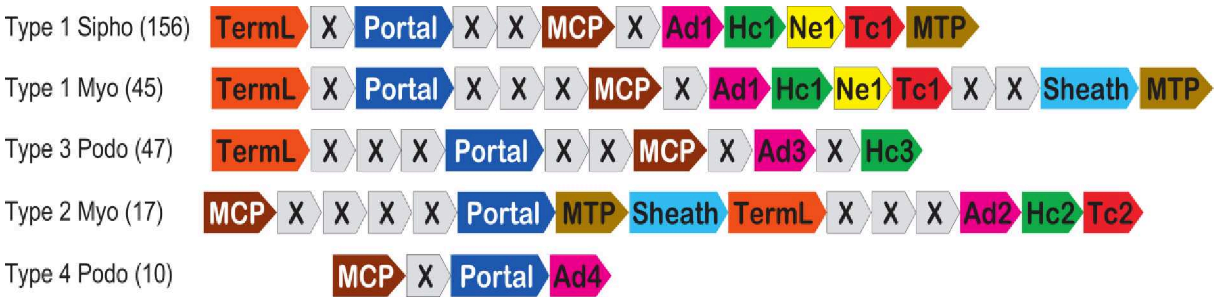

## Supplementary figure 2

## Secondary structure analysis of Type 1 neck Ne1 proteins identified in (A) *Siphoviridae* and (B) *Myoviridae.*

Profile-profile comparison using HHsearch provided sequence alignments for Ne1 proteins. Because of the high sequence divergence of these proteins, only alignments of the predicted secondary structure elements are presented for a set of Ne1 proteins (identified by their Genbank Identifyers). This set of proteins is representative of most detected Ne1 proteins. Indeed, Aclame classifies proteins into families of homologs. In (A), patterns representative of the 5 Aclame Ne1 families comprising more than 10 proteins are displayed, together with the secondary structure patterns of the shortest (4604) and longest (6375) Ne1. In (B), patterns representative of the 3 Aclame Ne1 families comprising more than 5 proteins are displayed, together with the secondary structure patterns of the shortest (102338) and longest (3787) Ne1. All the displayed patterns (except that of 4604) correspond to proteins detected as analogous to SPP1 gp16.1 by HHSEARCH using a confidence threshold of 95%. Protein 4604 was detected with a confidence threshold of 80%. Correspondence between the Aclame identifiers found on this figure and the GenBank identifiers is the following (using the same order): in fig. A, 9635175, 22296530, 118430771, 71834102, 41189542, 48697267, 91214209 and in fig. B, 90592648, 84662658, 9634069, 17975132, 17981836.

**(A)**

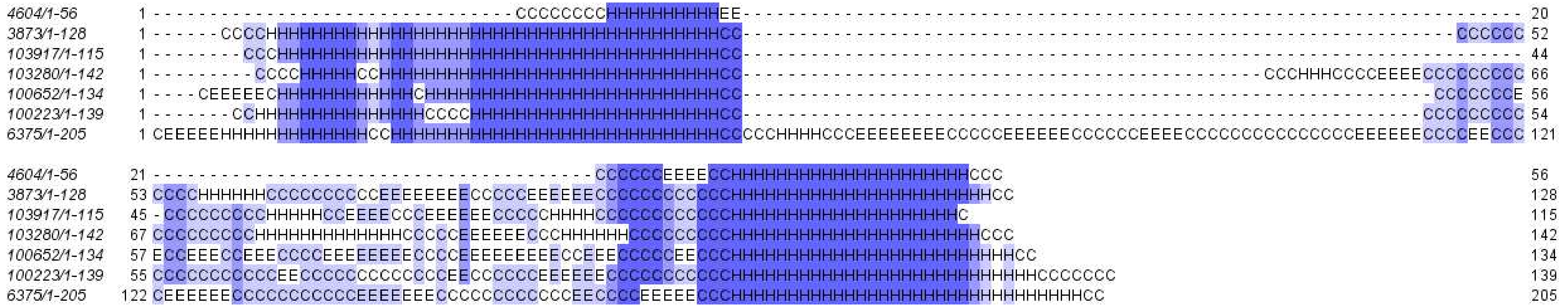


**(B)**

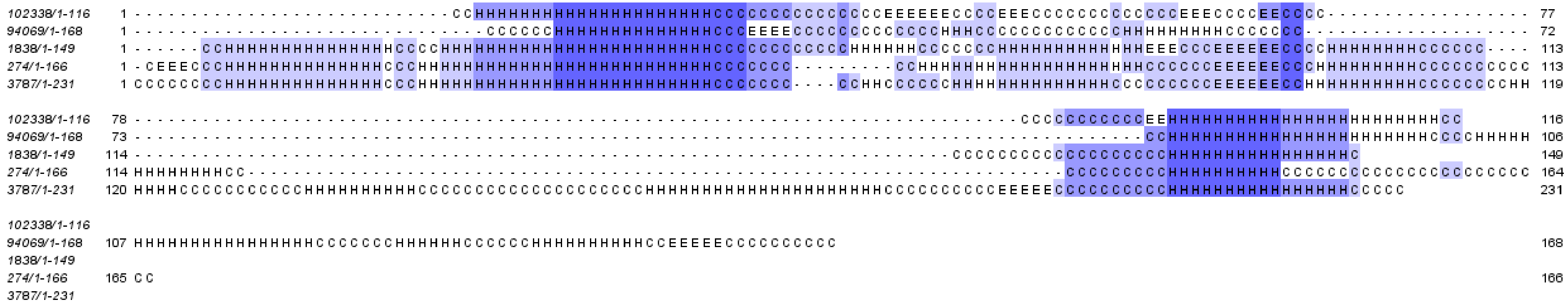

**Supplementary figure 3**

**Matrices of profile-profile comparison and identity scores (A) within a typical Type 1 Cluster (Cluster 1) and (B) between Type 1 Clusters**

(A) Analysis of Cluster 1 phage neck homologies. HHsearch probability scores are plotted above the diagonal and sequence identity percentages are plotted below the diagonal. Both use the following legend:

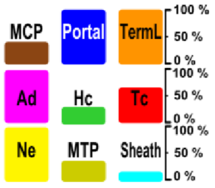


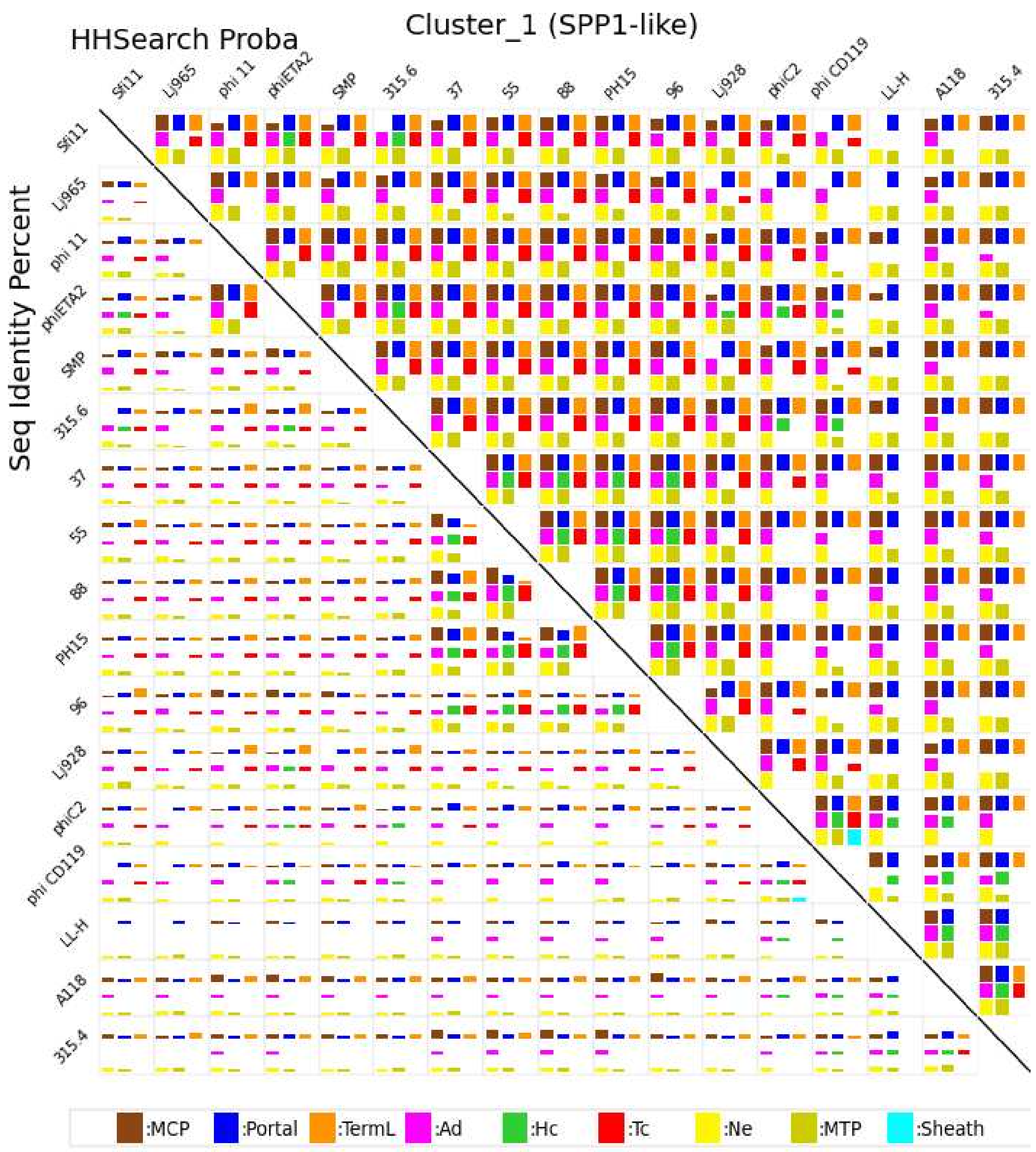

(B) Analysis of phage neck homologies between Clusters. HHsearch probability scores are plotted using the same codes as in (A).

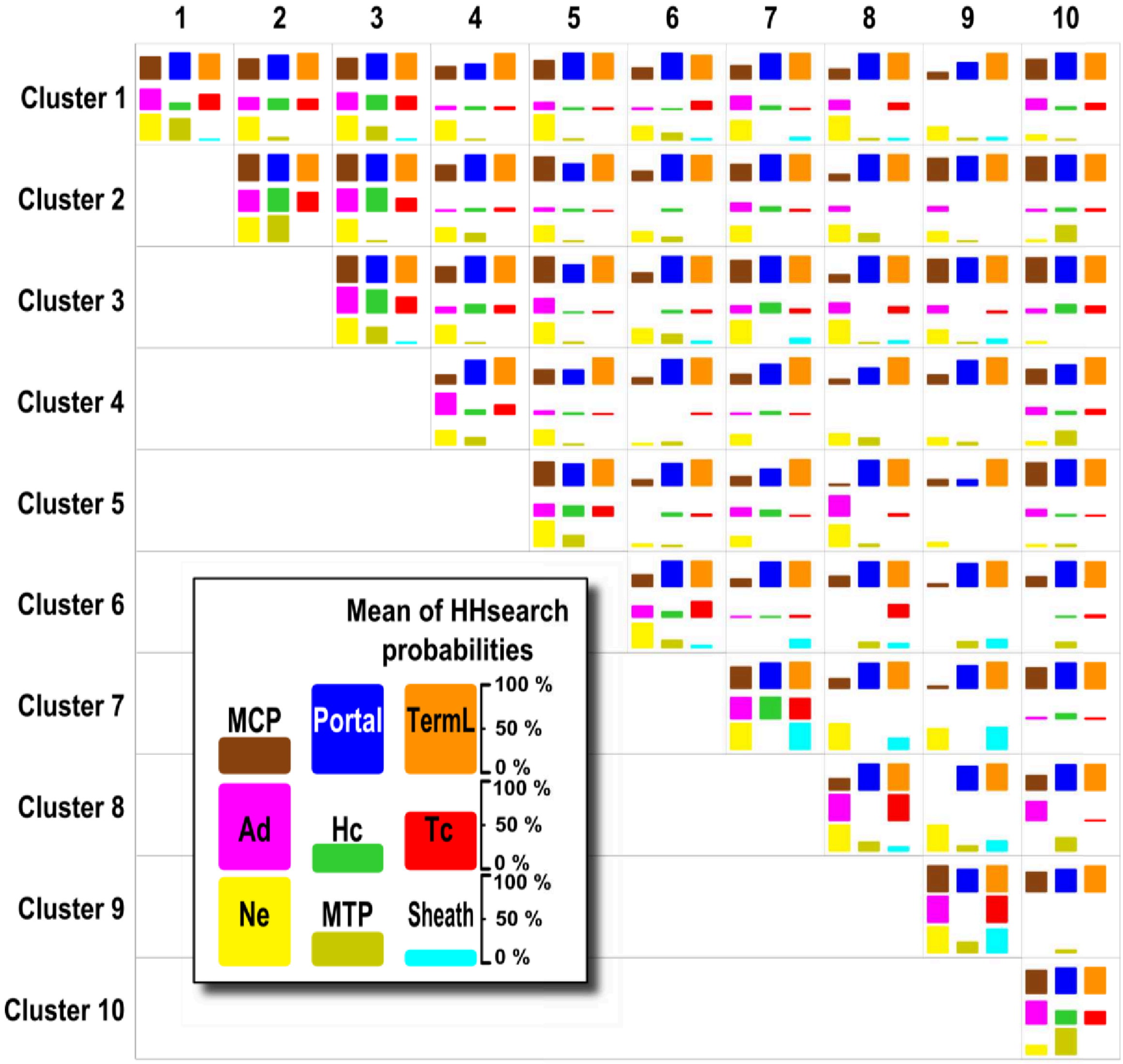

**Supplementary Figure 4**

Distribution of phage types and clusters over the different bacterial clades organised using the circular tree defined in (Ciccarelli FD, Doerks T, von Mering C, Creevey CJ, Snel B, Bork P. *Science.* (2006) 311(5765):1283-7; Letunic I, Bork P. *Nucleic Acids Res.* (2011) 39:W475-8.) . Names of the bacterial clades are indicated in the inner circle with pink and cyan arches spanning the different bacterial species. The coverage of the clusters and types are indicated by grey arches labelled using colored square boxes in the outer circle.

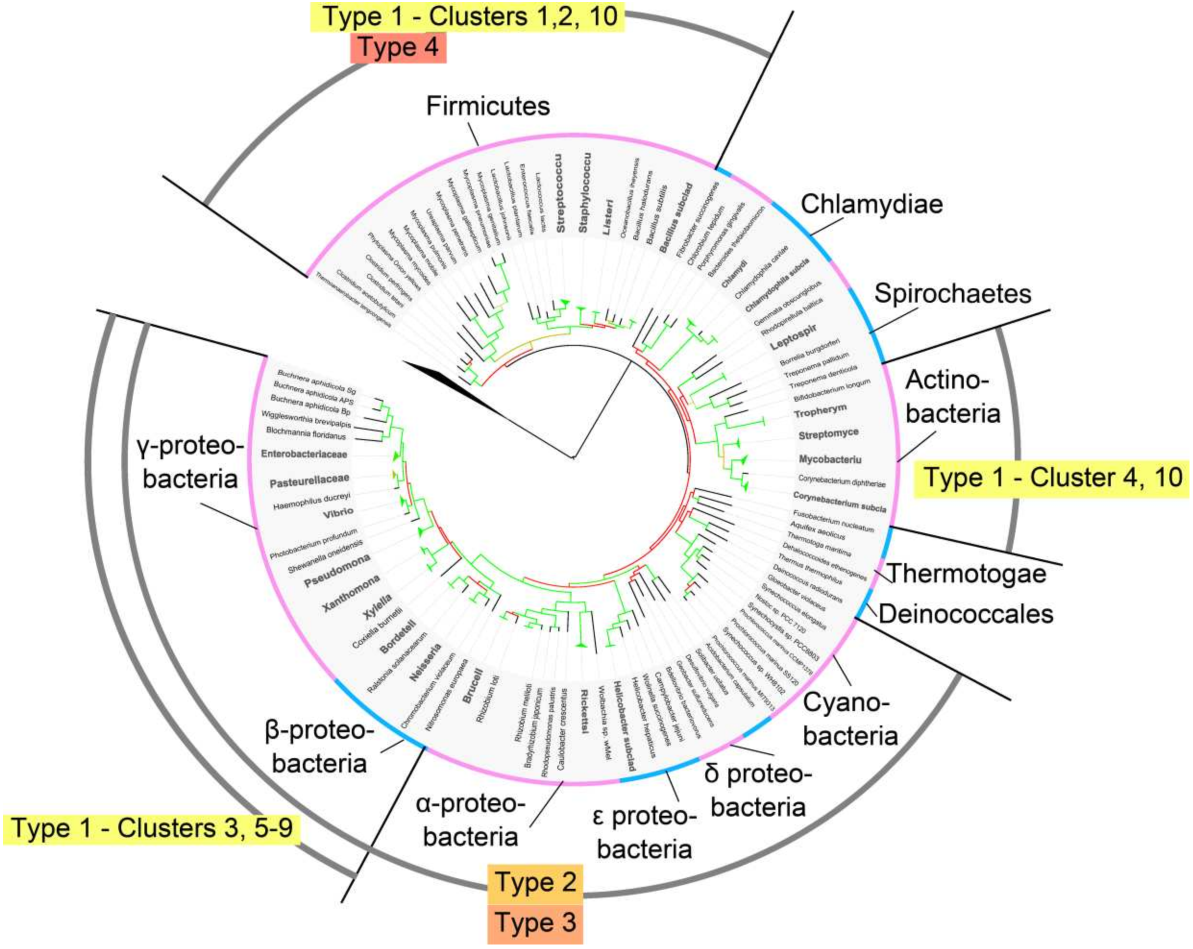

**Supplementary Figure 5**

Global trees representing the global classification of phages from Aclame when only **(A)** the the Portal proteins or **(B)** the MCP-TermL-Portal triad proteins were used to calculate the similarity matrix between phages. The similarity scores were calculated using the same metric as described in the manuscript combining HHsearch probabilities and sequence identities. The background colors report for the Clusters and Types that were defined using the multi-proteins classification. Along the external circle, small boxes indicate the cluster assignment for every phage (T1.x stands for "Type 1 Cluster x", T2 for Type 2, etc… ). A colored star was added outside the circle to pinpoint inconsistent grouping of some phages outside the Clusters defined from the multi-proteins classification.

The inconsistencies were rated using three criteria. First, we controlled whether the four types are properly discriminated. Second, we analyzed the clusters generated and whether the phages that are grouped together shared the same host categories (Firmicutes/Proteobacteria/Actinobacteria) as in Figure 3. Third, we also used as sensitivity markers pairs of phages such as N15 (Siphoviridae)/ VP882 (Myoviridae), grouped together in Cluster 6 (Type 1). These two phages, although quite distant, share the same biological properties of a linear genome containing a protelomerase gene, suggesting that they belong to a group diverged from a common ancestor [1]. Other methodologies were tested to produce the global tree with the single Portal marker : a multiple sequence alignment of all Portal sequences using Mafft linsi algorithm, trimming or not the gaps, using either Neighbour-Joining algorithm or more advanced methodologies included in the PhyML method to calculate the tree. In none of these cases, the derived classification was better than the one produced using the HHsearch-based similarity matrix shown in Suppl. Fig. 5A.

Altogether, our metrics supports that MCP, TermL and Portal are indeed informative and that Portal is probably the best single protein marker to classify phages. However, Portal can also diverge in evolution while head-to-tail connection proteins still show consistent evolutionary links with proteins from other phages. We have added a new Figure, Figure 5, to illustrate this observation. To appreciate the protein sequence relationships between these phages, it is important to remind that above the so-called "twilight zone" threshold in the 30-35% identity range, common evolutionary history can be fairly well established. Below that threshold, sequence identity is generally assumed to be a less accurate proxy for reporting homology

relationships. Below 20%, lies the “remote zone” in which sequence identity is no longer a useful proxy for detecting common evolutionary history. Many phages have Portal sharing less than 30% identity with other Aclame proteins. Despite this diversity, it is remarkable that a majority of phages with divergent Portal can still be correctly clustered in the Portal-only tree. We believe that this arises from the fact that there is a dense reticulation of similar phages, which tend to properly attract phages with divergent Portal to the correct cluster of related phages. However, in case reticulation between similar phages is low (as for phages from Actinobacteria in Clusters 4 and 10), Portal may not suffice to drive proper clustering. In that case, markers physically connected to the head-neck-tail module (and in particular head-to-tail connection markers) may turn as a useful proxy to favor the clustering of evolutionary related phages. The metric used to establish the phage similarity matrix account for that property. As soon as a protein pair will have significant match above 30% it will contribute favorably to phage association. The fact that our 4 Types multi-proteins classification is (i) globally consistent with Portal-only classification (ii) successful in difficult cases in recognizing phages hosted in Bacteria of the same phylum supports the idea that neck proteins bring useful signal to the classification of phages. We speculate that because Portal and neck proteins have direct physical contacts, their evolution was sufficiently correlated so that the signals from the neck proteins do not add too much noise to the classification and rather helps recognizing related phages when for some reasons Portal sequences are more divergent.

1. Ravin NV: N15: the linear phage-plasmid. Plasmid 2011, 65(2):102-109.

Supplementary Figure 5A

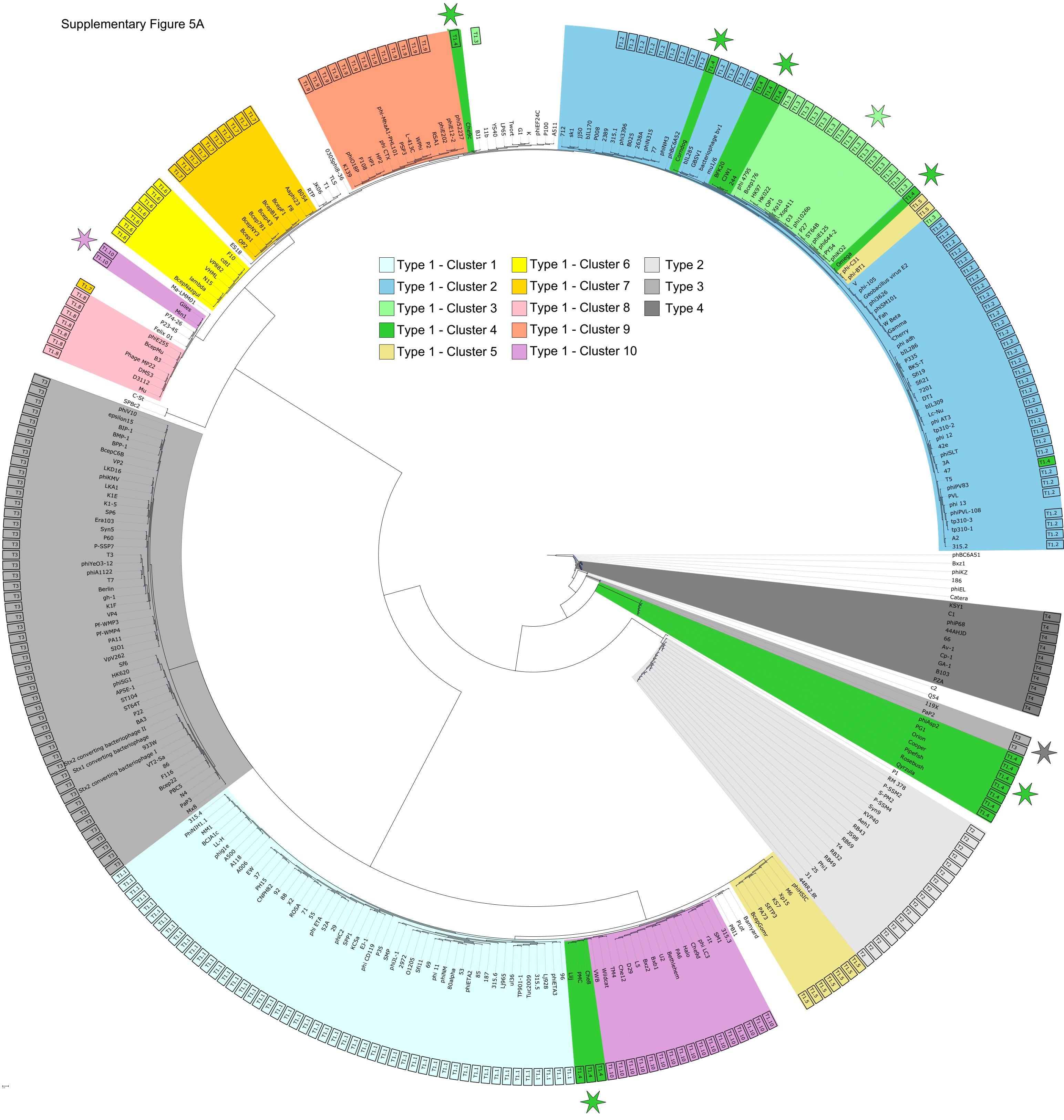

Supplementary Figure 5B

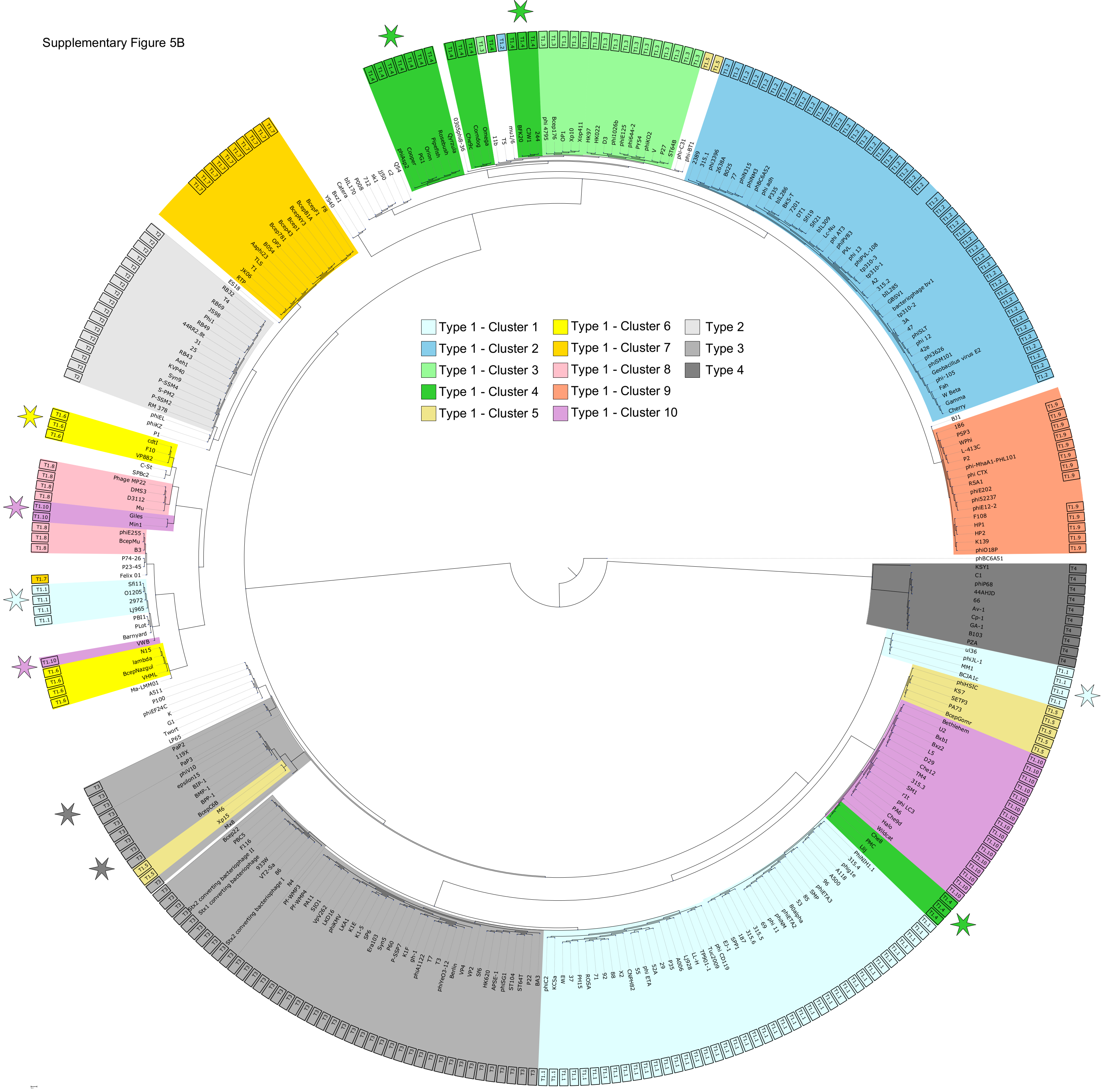

**Supplementary Figure 6**

Tree representations of phage similarities, built from a hierarchical agglomerative clustering procedure applied to a matrix of similarity scores between pairs of phages.

(A) Type 2 phages

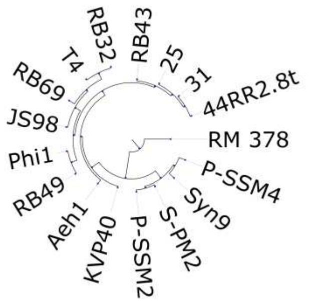


(B) Type 3 phages

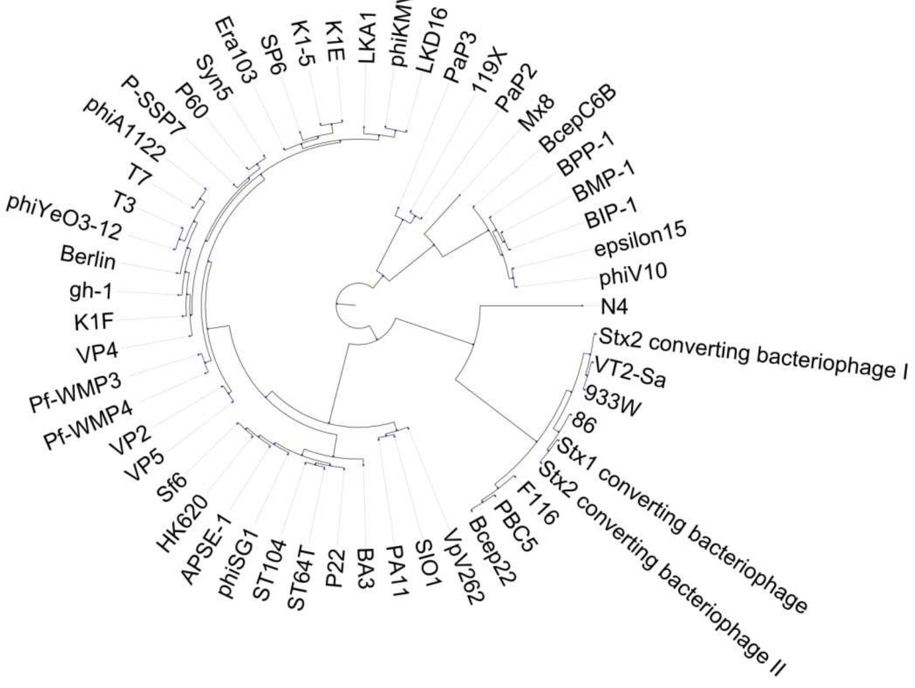


(C) Type 4 phages

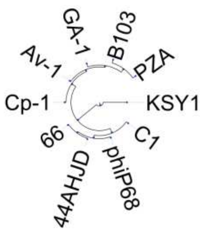

**Supplementary figure 7:**
**Distribution of the Aclame phages as a function of their number of genes.**

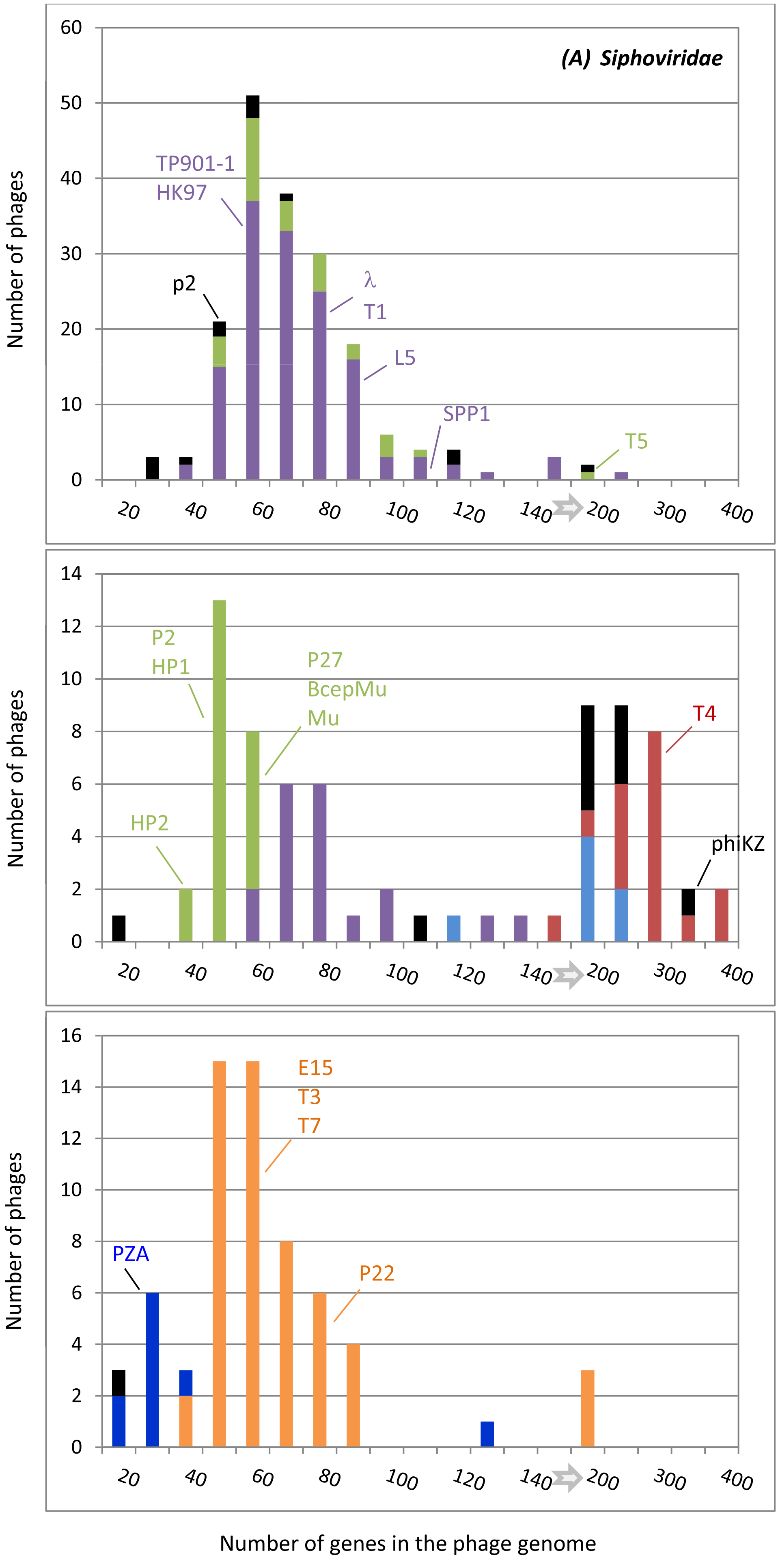


**Color code:**

Magenta : Type 1 with 4 detected head-to-tail completion genes

Green : Type 1 with 2-3 detected head-to-tail completion genes

Cyan : Type 1 with only Ad1 detected

Red: Type 2

Orange: Type 3

Blue: Type 4

Black: not assigned by our approach

**Supplementary Text 1 : Relationship between neck structural organisation and genome size in tailed bacteriophages**

In our study, *Siphoviridae* represent a homogeneous phage family that generally exhibits a SPP1-like (Type 1) neck and have between 31 and 150 genes (Suppl. Fig. 4A). They share the neck structural organisation of phages SPP1, TP901-1, HK97, λ, T1 and L5 (Maxwell et al. 2001; Maxwell et al. 2002; Edmonds et al. 2007; Lhuillier et al. 2009; Cardarelli et al.). The large majority of these phages exhibit 4 detected head-to-tail connection proteins (Table 3). Twenty seven siphophages (16%) have only 3 head-to-tail connection proteins and for 1 phage only 2 of these proteins were detected. The missing protein is often Hc1, as observed in T5 and 21 other phages. In these phages, a completely different Hc protein may be used, or the stopper function may be performed by Ad1, even if no clear structural difference could be identified between Ad1 of phages with or without Hc1.

Most myophages belong to two structural groups that adopt either SPP1-like (Type 1) or T4-like (Type 2) neck structures. These groups drastically differ by the size of their genomes (Suppl. Fig. 4B). *Myoviridae* with a genome size similar to that of *Siphoviridae* (between 31 and 150 genes) generally exhibit a SPP1-like neck. Moreover, most of the smallest phages (between 31 and 60 genes; ex: HP1, HP2, P2, P27, BcepMu, Mu) are deprived of Hc1, while the other phages (between 61 and 150 genes; ex: AaΦ23) have all four Ad1, Hc1, Tc1 and Ne1 components (Suppl. Fig. 4B). The neck structural organisation of the largest *Myoviridae* (between 151 to 381 genes) is in sharp contrast with that of small *Myoviridae*. Half of the large myophages have a Type 2 neck comprising the Ad2, Hc2 and Tc2 trio (Suppl. Fig. 4B), while for the other half our methods could not detect any annotated head-to-tail connection proteins prompting for further exploration of these unknown systems.

*Podoviridae* also belong to two structural groups that adopt P22-like (Type 3) and Φ29-like (Type 4) neck structures, respectively. Here again, these groups drastically differ by the size of their genomes (Suppl. Fig. 4C). *Podoviridae* with a genome size similar to that of siphophages (between 31 to 150 genes) generally exhibit a P22-like neck: they adopt a neck structural organisation similar to that described for P22 (ex: T3, T7, ε15) (Olia et al. 2011; Tang et al. 2011). Tiny phages (containing less than 30 genes) generally present a Φ29-like neck (ex: PZA, c1).

**Supplementary Text 2 : Structural analogies between the different neck Types**

Our HHsearch analysis revealed structural relationships within each functional category: Ad, Hc and Tc. Analysing adaptor proteins, we observed that Ad1, Ad2, Ad3 and Ad4 are all predicted to contain 4 to 5 $\alpha$-helices. For one particular Ad1-Ad3 pair, HHsearch strongly predicts homology: the Ad1 of *Siphoviridae* phage Che9c and the Ad3 of *Podoviridae* phage 933W, have profiles matching with a probability value of 81%. Experimental evidence, derived from the structural characterisations of the Ad1 gp15 from SPP1 (Lhuillier et al. 2009), gp6 from HK97 (Cardarelli et al. 2010) and of the Ad3 gp4 from P22 (Olia et al. 2011) consistently revealed that Ad1 and Ad3 proteins shared the same $\alpha$-helical bundle fold. Examination of Ad1 to Ad3 profile alignments suggest that these two groups are segregated on the basis of large insertions within loops $\alpha1\alpha2$ and $\alpha2\alpha3$ and at the C-terminus. In a similar way, Hc1, Hc2 and Hc3 are all predicted to fold into a $\beta$–strand rich structure. Furthermore, one match was detected between a Hc1 (*Siphoviridae* phage M6) and a Hc2 (*Myoviridae* phage Syn9) with a probability value of 71%, suggesting that these two superfamilies could share a structural core. Finally, the tail-completion proteins Tc1 and Tc2 both adopt an $\alpha$-$(\beta)_n$-$\alpha$-$(\beta)_n$ structure. Here again, a match was observed between a Tc1 (*Siphoviridae* phage BCJA1c) and a Tc2 (*Myoviridae* phage 25) with a probability value of 77%. The very recent determination of the 3D structure of a Tc2 protein (gp15 from phage T4) consistently revealed its structural homology with a Tc1 protein, gpU from phage $\lambda$ (Fokine et al., 2013). Altogether, these observations support the existence of extended structural homologies between the different neck architectures. Additional experimental results are now needed in order to further describe a potential common structural core at the phage head-to-tail connection.

**Supplementary Method 1 : Procedure to assess the sensitivity of PSI-Blast search for Table 2**

To compare the capacity of PSI-Blast to retrieve remote homologs with respect to that of HHsearch, we first defined, for every neck component, a set of reference profiles that could then be used as queries against the database of 28300 sequences contained in Aclame. These reference profiles were retrieved from two profiles databases, PFAM and CDD (Marchler-Bauer et al. 2013; Finn et al. 2014). To obtain the comprehensive list of reference profiles reported in Supp. Table 3, we actually used HHsearch and queried the PFAM and CDD profiles with the profiles of every neck proteins identified in our work. The reference PFAM and CDD profiles were in turn used as queries of a PSI-Blast search against all the 28300 sequences of the proteins contained in Aclame. All the proteins matched by this protocol were enumerated and their numbers are reported in Table 2.

**References**


Cardarelli L, Lam R, Tuite A, Baker LA, Sadowski PD, Radford DR, Rubinstein JL, Battaile KP, Chirgadze N, Maxwell KL et al. 2010. The crystal structure of bacteriophage HK97 gp6: defining a large family of head-tail connector proteins. *J Mol Biol* **395**(4): 754-768.

Edmonds L, Liu A, Kwan JJ, Avanessy A, Caracoglia M, Yang I, Maxwell KL, Rubenstein J, Davidson AR, Donaldson LW. 2007. The NMR structure of the gpU tail-terminator protein from bacteriophage lambda: identification of sites contributing to Mg(II)-mediated oligomerization and biological function. *J Mol Biol* **365**(1): 175-186.

Finn RD, Bateman A, Clements J, Coggill P, Eberhardt RY, Eddy SR, Heger A, Hetherington K, Holm L, Mistry J et al. 2014. Pfam: the protein families database. *Nucleic Acids Res* **42**(Database issue): D222-230.

Lhuillier S, Gallopin M, Gilquin B, Brasiles S, Lancelot N, Letellier G, Gilles M, Dethan G, Orlova EV, Couprie J et al. 2009. Structure of bacteriophage SPP1 head-to-tail connection reveals mechanism for viral DNA gating. *Proc Natl Acad Sci U S A* **106**(21): 8507-8512.

Marchler-Bauer A, Zheng C, Chitsaz F, Derbyshire MK, Geer LY, Geer RC, Gonzales NR, Gwadz M, Hurwitz DI, Lanczycki CJ et al. 2013. CDD: conserved domains and protein three-dimensional structure. *Nucleic Acids Res* **41**(Database issue): D348-352.

Maxwell KL, Yee AA, Arrowsmith CH, Gold M, Davidson AR. 2002. The solution structure of the bacteriophage lambda head-tail joining protein, gpFII. *J Mol Biol* **318**(5): 1395-1404.

Maxwell KL, Yee AA, Booth V, Arrowsmith CH, Gold M, Davidson AR. 2001. The solution structure of bacteriophage lambda protein W, a small morphogenetic protein possessing a novel fold. *J Mol Biol* **308**(1): 9-14.

Olia AS, Prevelige PE, Jr., Johnson JE, Cingolani G. 2011. Three-dimensional structure of a viral genome-delivery portal vertex. *Nat Struct Mol Biol* **18**(5): 597-603.

Tang J, Lander GC, Olia AS, Li R, Casjens S, Prevelige P, Jr., Cingolani G, Baker TS, Johnson JE. 2011. Peering down the barrel of a bacteriophage portal: the genome packaging and release valve in p22. *Structure* **19**(4): 496-502.

**Supplementary Table 1 :** The table is provided in the excel file SupplementaryTable1_Lopes_etal.xlsx

**Supplementary Table 2 :** The table is provided in the excel file SupplementaryTable2_Lopes_etal.xlsx

**Supplementary Table 3** : List of sequence profiles extracted from PfamA and CDD databases matching phage neck proteins from the four defined Types. These profiles were used to monitor the sensitivity of PSI-Blast for identifying remote homologs of the neck proteins from the phage of the aclame database and compare it to the performances of profile-profile search strategies (reported in Table 2).

| | |
|---|---|
| Ad1 | cdd_cd08051;cdd_cd08053;cdd_cd08054;cdd_cd08055;cdd_TIGR01560;cdd_TIGR02215; pfamA_PF05135; pfamA_PF11436; pfamA_PF02831; pfamA_PF05926; pfamA_PF13262 |
| Ad2 | cdd_PHA02554 |
| Ad3 | pfamA_PF11650 |
| Ad4 | cdd_PHA00148; cdd_PHA01077 |

| | |
|---|---|
| Hc1 | cdd_PHA02310 ; cdd_TIGR01563; pfamA_PF05521; pfamA_PF10665; pfamA_PF05354; pfamA_PF13856 |
| Hc2 | cdd_PHA02555; pfamA_PF11649 |
| Hc3 | cdd_PHA00661; pfamA_PF11134 |

| | |
|---|---|
| Tc1 | pfamA_PF11367; pfamA_PF06141; pfamA_PF11367; pfamA_PF13554; pfamA_PF06891; cdd_PF06141; cdd_PF11367; cdd_PF13554; pfamA_PF06141; pfamA_PF11367; pfamA_PF13554 |
| Tc2 | cdd_PHA02556 |
